# Elastic pseudospin transport for integratable topological phononic circuits

**DOI:** 10.1038/s41467-018-05461-5

**Published:** 2018-08-06

**Authors:** Si-Yuan Yu, Cheng He, Zhen Wang, Fu-Kang Liu, Xiao-Chen Sun, Zheng Li, Hai-Zhou Lu, Ming-Hui Lu, Xiao-Ping Liu, Yan-Feng Chen

**Affiliations:** 1 National Laboratory of Solid State Microstructures & Department of Materials Science and Engineering, Nanjing University, Nanjing, 210093 China; 2 Collaborative Innovation Center of Advanced Microstructures, Nanjing University, Nanjing, 210093 China; 3 Jiangsu Key Laboratory of Artificial Functional Materials, Nanjing, 210093 China; 4 Shenzhen Institute for Quantum Science and Engineering and Department of Physics, South University of Science and Technology of China, Shenzhen, 518055 China

## Abstract

Precise control of solid-state elastic waves’ mode content and coherence is of great use nowadays in reinforcing mechanical energy harvesting/storage, nondestructive material testing, wave-matter interaction, high sensitivity sensing, and information processing, etc. Its efficacy is highly dependent on having elastic transmission channels with lower loss and higher degree of freedom. Here, we demonstrate experimentally an elastic analog of the quantum spin Hall effects in a monolithically scalable configuration, which opens up a route in manipulating elastic waves represented by elastic pseudospins with spin-momentum locking. Their unique features including robustness and negligible propagation loss may enhance elastic planar-integrated circuit-level and system-level performance. Our approach promotes topological materials that can interact with solid-state phonons in both static and time-dependent regimes. It thus can be immediately applied to multifarious chip-scale topological phononic devices, such as path-arbitrary elastic wave-guiding, elastic splitters and elastic resonators with high-quality factors.

## Abstract (web summary)

Precise control of elastic waves is of great use in current technologies. Here, Yu et al. realize the analogue of quantum spin Hall effects for the elastic waves in a plain plate consisting of identical perforated holes in wavelength scales.

## Introduction

Phonons play a fundamental role in modern industrial civilization, for they are elaborately used to carry and process information with great accuracy in time, frequency and phase domains in a wide variety of fields. It is especially the case in the solid-state elastic world, since elastic phonons simultaneously possess several crucial advantages compared with fluid/airborne ones, such as: scalability towards integrated devices, antijamming capability, energy capacity, and extremely low losses. Elastic phonons found in solids are nowadays key to high signal-to-noise ratio information processing^1^, high-sensitive and remote sensing, nondestructive testing of the internal structure of matters as well as intense wave-matter interaction for future quantum acoustics^2^. Fully unleashing the potential of these phonons, however, requires a precise manipulation of their temporal as well as spatial dynamics (i.e., waveguiding), which is often not so trivial compared with other particles (electrons, photons, or fluid phonons). Acoustic impedances of solid-state materials are relatively high and close, so it is basically ineffective in realizing conventional index guiding with large impedance contrast for elastic phonons in any continuous medium. Worse still, defects in solids are much more ubiquitous, further aggravating elastic dissipation induced by scattering of the phonons. Hence, it will be highly welcome to have a material that provides practically viable means of manipulating elastic transport dynamics, thereby offering a high degree of freedom in elastic waveguiding along with low transmission losses.

Topological states have been discovered in various electronic systems in past decades. Recently, the core principle behind these states has been adapted to bosonic systems, leading to the discovery and demonstration of topological photonics^3–17^ and mechanics^18^ (in both platforms built from discrete/weakly-interacting man-made atoms^19–22^ and fluid acoustic systems^23–27^). An essential impetus of these explorations is about gapless edge states, for they can be transported in a backscattering-immune fashion with unparalleled tolerance towards any “non-magnetic” defects, arbitrary bends, and fabrication imperfections. This performance superiority thus has promoted an era of functional large-scale topological photonic circuits^28–30^.

Topological states (in particular, the quantum Hall family) for elastic waves (specifically, linear stress waves in continuous solids) have been predicted in theoretical works^31–33^ using, e.g., gyroscopic inertial effects to break the time-reversal (TR) symmetry^31^ and sub-wavelength meta-structures to create effective spins^32^. None of them have been demonstrated experimentally, mainly due to herculean engineering difficulties of these material phases. To date, almost all the preceding acoustic topological phases are realized in the only scalar (longitudinal wave) system of fluid airborne sound, whose practical role is largely limited. It is thus not only a remaining intellectual challenge but also of great practical value to realize the elastic topological states, especially in a generally concise configuration that can be scaled accordingly for future chip-scale applications.

Here, we demonstrate an elastic quantum spin Hall effect (QSHE) in probably the simplest solid-state structure, i.e., a plain plate consisting of identical perforated holes in wavelength scales. The structural material possesses an elastic accidentally double Dirac cone with fourfold degeneracy. These four degenerate modes can be hybridized to form two elastic pseudospin-½ and then mimicking QSHE via geometric tuning. The topologically protected elastic transport against structural imperfections and disorders has allowed us to demonstrate several unprecedented functional components for elastic phonons. Examples include arbitrary elastic pathway, elastic beam splitter, and high-quality elastic resonator in arbitrary geometries. These demonstrated high-performance components may serve as building blocks for the coming era for large-scale phononic circuits and networks (e.g., elastic interferometers), paving the way for advance acoustic signal processing, sensing and even analogue computing. Our configuration can be fully scaled down in size to operate in RF region, boosting the performance of and/or adding new functionality into the existing integrated surface/bulk acoustic wave devices.

## Results

### Elastic pseudospin-½ via an accidental fourfold degeneracy

**Fig. 1:**
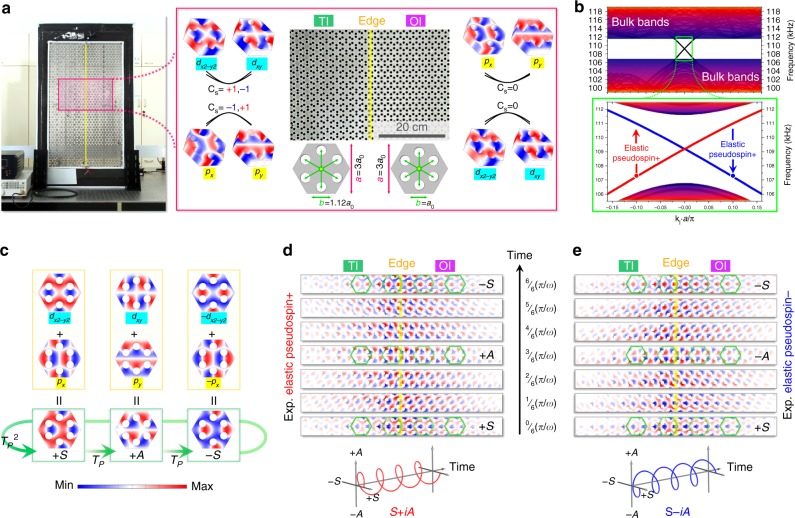
Elastic pseudospins and helical edge states. **a** Topologically protected interface (yellow lines) formed by two different elastic insulators (perforated phononic crystal on the same plates). These two elastic insulators are identical in lattice constant *a* (3*a*_0_), plate thickness ($\sqrt 3\,\times\,$0.4*a*_0_), and radius of perforated holes *r* ($\sqrt 3\,\times\,$0.18*a*_0_) but different hole-center distance characterized by *b*. When *b* is reduced from (right) *b* = 1.0*a*_0_ to (left) *b* = 1.12*a*_0_, the resulted band diagram evolution indicates the occurrence of a band inversion process for the *p*_*x*_/*p*_*y*_ and *d*_*x2−y2*_/*d*_*xy*_ modes (similar to *p*/*d* orbitals of electrons), corresponding to a topological transition from an ordinary insulator [OI, zero spin Chern number (Cs)] to a topological insulator [TI, none-zero Cs] with an overlapped bulk bandgap. **b** Projected band diagram of the TI–OI interface and a zoomed-in view illustrating two elastic helical edge states (characterized by two elastic pseudospins). The grey dispersions correspond to shear-horizontal waves and are not excited in our study. **c** Out-of-plane displacements of the degenerate modes indicating their evolutionary relationship: bulk *p* and *d* modes hybridize to form a pair of normal modes, i.e., one symmetric mode $S = \left( {p_x + d_{x2 - y2}} \right)/\sqrt 2$ and one anti-symmetric mode $A = \left( {p_y + d_{xy}} \right)/\sqrt 2$, used as the basis to construct the two pseudospins, *S* + i*A* and *S* *−* i*A*, protected by the pseudo (fermi-like) time reversal symmetry (*T*_*p*_^2^ = −1), as $+ {\it{S}}\mathop{\longrightarrow}\limits^{{{\it{T}}_{\it{p}}}} + {\it{A}}\mathop{\longrightarrow}\limits^{{{\it{T}}_{\it{p}}}} - {\it{S}}\mathop{\longrightarrow}\limits^{{{\it{T}}_{\it{p}}}} \cdot \cdot \cdot$. **d,** **e** Experimentally recorded temporal evolution of elastic field distribution of the out-of-plane displacement in the vicinity of the TI–OI interface when the elastic wave is excited from the bottom of the interface (upward-traveling) and from the top (downward-traveling), respectively. These time-domain results in a half period (from *t* = 0 to *t* = *π/ω*) strongly confirm the existence of two spin-momentum locked elastic pseudospins represented in the *S*/*A* basis: time-dependent anti-clockwise elastic pseudospin + (i.e., *S* + i*A*) and clockwise elastic pseudospin− (i.e., *S* − i*A*), respectively

In bosonic systems, the most common methods to break TR symmetry is using a gauge field, e.g., a magnetic field for photons^34^ or an effective gauge field generated with a dynamic modulation for phonons^35,36^/photons^37^. However, in order to obtain TR-invariant bosonic QSHE, a totally different approach is needed. TR operator of bosons (*T*_*b*_^2^ = + 1) is essentially distinct from that of fermions (*T*_*f*_^2^ = −1), meaning that bosonic systems cannot naturally guarantee Kramers degeneracies, a prerequisite for QSHE. Consequently, the quest of realizing bosonic QSHE solely relies on the construction of artificial bosonic spin-½ states (herein referred as pseudospins) under a pseudo (fermi-like) TR symmetry (*T*_*p*_^2^ = −1). Generally, these pseudospins can be emulated through polarization or modal hybridization. For instance, two degenerate modes *M*_1_ and *M*_2_ can be hybridized to construct these pseudospins: spin+/− ≡ *M*_1_ + i*M*_2_/*M*_1 _– i*M*_2_, if only ${\it{M}}_{\mathrm{1}}\mathop{\longrightarrow}\limits^{{{\it{T}}_{\it{p}}}}{\it{M}}_{\mathrm{2}}$ while ${\it{M}}_{\mathrm{2}}\mathop{\longrightarrow}\limits^{{{\it{T}}_{\it{p}}}} - {\it{M}}_{\mathrm{1}}$. In our elastic plate, an elastic fourfold degeneracy can be accidentally formed [see details in Supplementary Note 1], further leading to the pseudospins for elastic waves under the pseudo TR symmetry, as exemplified in Fig. 1.

In a single plate (Fig. 1a), two different 2D elastic insulators (plate phononic crystals with elastic bandgap) are fabricated next to each other, forming an interface. These insulators have the same point group (*C*_6*v*_) and lattice constant *a*. The only difference between them is the hole-center distance *b* (measured from the center of the six perforated holes to the center of the unit-cell). The elastic insulator on the right side of the interface has a relatively small *b* (equals to *a*_0_, where 3*a*_0_ = *a*), and it supports two pairs of twofold degeneracy at the Brillouin zone center (**k** = 0), forming an elastic bandgap. The degeneracy at the band edges corresponds to *p*_*x*_/*p*_*y*_ (-like) bulk modes at higher frequencies and *d*_*x2−y2*_/*d*_*xy*_ (-like) bulk modes at lower frequencies, respectively, which are similar to *p* and *d* orbitals of electrons. Here *p*_*x*_ obeys symmetry *σ*_*x*_*/σ*_*y*_ = −1/+1; *p*_*y*_ obeys + 1/−1; *d*_*x2*−*y2*_ obeys + 1/+1; and *d*_*xy*_ obeys −1/−1, where *σ*_*x(y)*_ = +1, −1 represents the even or odd symmetry along the *x* or *y* axis, respectively. The other elastic insulator on the left has a relatively large *b* (equal to 1.12*a*_0_) resulting in an inversion of the *p* and *d* modes at the band edges. In between these two cases, there exists a configuration (*a*_0_ < *b*≈1.0873*a*_0_ < 1.12*a*_0_), where the elastic bandgap completely vanishes, leading to a double Dirac point with the fourfold degeneracy. i.e., a topological transition point between an ordinary insulator (OI) and a topological insulator (TI, see Supplementary Note 2) with an overlapped bulk bandgap.

Projected band structure of the OI–TI interface is calculated and shown in Fig. 1b. Ignoring the dispersion of shear-horizontal modes (SH modes, see more in Supplementary Note 3) denoted by grey lines, a clean single Dirac cone, formed by two gapless dispersions, appears at the Brillouin zone center (**k**_‖_ = 0) inside the overlapped bulk bandgap. These two gapless dispersions represent two helical edge states, originated from the degenerate *p* and *d* bulk modes of the two elastic insulators. As demonstrated in Fig. 1c, bulk *p* and *d* modes hybridize to form a pair of normal modes, i.e., one symmetric mode $S = ( {p_x + d_{x2 - y2}} ){\mathrm{/}}\sqrt 2$ and one anti-symmetric mode $A = ( {p_y + d_{xy}} ){\mathrm{/}}\sqrt 2$. Then, these two normal modes are used as the basis to construct the required two pseudospins, *S* + i*A* and *S* *−* i*A*, which are protected by the pseudo TR symmetry (*T*_*p*_^2^ = −1), as validated by placing the *T*_*p*_ operator on the *S*/*A* basis, i.e., $+ {\it{S}}\mathop{\longrightarrow}\limits^{{{\it{T}}_{\it{p}}}} + {\it{A}}\mathop{\longrightarrow}\limits^{{{\it{T}}_{\it{p}}}} - {\it{S}}\mathop{\longrightarrow}\limits^{{{\it{T}}_{\it{p}}}} \cdot \cdot \cdot$. Specific to our system, this *T*_*p*_ equals to $\left( {{\it{C}}_{\mathrm{6}} - \sigma _{\mathrm{v}} \cdot \sigma _{\mathrm{d}} \cdot {\it{C}}_{\mathrm{3}}} \right){\mathrm{/}}\sqrt 3$ (see more detailed visualized operation of *T*_*p*_ in Supplementary Note 1).

### Visualized elastic pseudospin transport with spin-momentum-locking

**Fig. 2:**
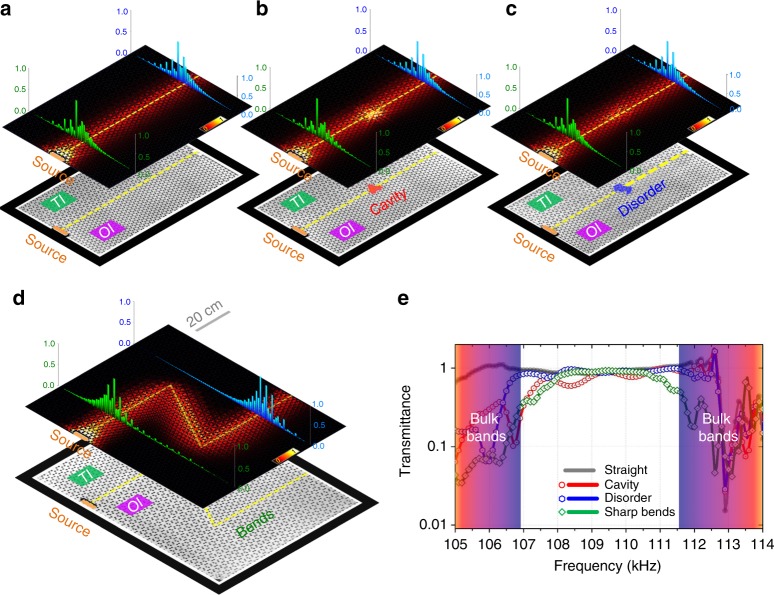
Flexible elastic pathways with backscattering immunity. **a**–**d** (bottom) Photographs of different topological-protected TI–OI interfaces to demonstrate robust and flexible elastic waveguiding. The yellow dotted lines indicate exact locations of the interfaces. TI–OI interfaces with **a** no defect and three different defects of **b** an arbitrary-shaped cavity without perforated holes, **c** an arbitrary-shaped disorder consisting of random ordered perforated holes **d** a *Z*-shaped bend. (middle) Heat maps are calculated elastic energy-density distributions for elastic waves at a frequency (110.0 kHz) within the bulk band-gap, corresponding to the four different configurations, respectively. (top) Green and blue bars are experimental results measured along two straight lines cut through the TI–OI interfaces before and behind the defects, respectively. **e** Experimental measured elastic transmission spectra for the four configurations. The black curve corresponds to the case without any defects as in **a**, while the red, blue, and green curves correspond to the case of **b**, **c**, and **d**, respectively. Shadow regions correspond to the bulk bands

These elastic pseudospins and the corresponding spin-momentum locked propagation at the TI–OI interface can be directly imaged by a laser vibrometer (see “Methods”). The propagating states at the interface are excited by an ultrasonic transducer placed on the surface of the plate. The amplitude of the out-of-plane displacement of the elastic wave [actually surface acoustic waves (SAWs)] is mapped out in the vicinity of the TI–OI interface. When excited from the bottom, as illustrated in Fig. 1d, the upward-traveling elastic wave exhibits a characteristic temporal evolution pattern, i.e., $+ {\it{S}} \to + {\it{A}} \to - {\it{S}} \to \cdot \cdot \cdot$. By contrast, when excited from the top, the downward-traveling elastic wave, as illustrated in Fig. 1e, exhibits also a characteristic but different pattern, i.e., $+ {\it{S}} \to - {\it{A}} \to - {\it{S}} \to \cdot \cdot \cdot$. In addition, regardless of the excitation conditions, the elastic energy is found to be highly located near the interface. All these dynamic visual results are conclusive evidences demonstrating 1) the existence of time-dependent anti-clockwise elastic pseudospin+ (i.e., *S* + i*A*) and the clockwise elastic pseudospin− (i.e., *S* − i*A*), as illustrated in the *S*/*A* normal mode basis (bottom insets of Figs. 1d, e and 2) edge state transport with spin-momentum locking at the TI–OI interface, i.e., pseudospin+ only in the upward-traveling direction while pseudospin− only in the downward-traveling direction, characterizing intuitively an elastic counterpart of QSHE.

Meanwhile, this spin-momentum locked elastic transport enables elastic pseudospin separation with arbitrary excitation states, which can be faithfully confirmed in an elastic pseudospin-selective waveguide coupler (i.e., an elastic beam splitter, see Supplementary Note 5).

### Integratable elastic phononic circuits with backscattering immunity

An ideal elastic waveguide can be constructed using our topologically protected TI–OI interface. Due to the spin-momentum-locking characteristic of the helical edge states, the backscattering of the elastic edge propagating modes (Mode_+**k**_$\mathop{\longleftrightarrow}\limits^{{{\mathrm{scattering}}}}$Mode_−**k**_) is intrinsically suppressed, making this elastic waveguide robust against various defects and distortions and mostly importantly allowing for a flexible geometry configuration of its pathways.

Experimentally, different defects are intentionally introduced into our TI–OI interfaces (elastic waveguides) to study the elastic wave propagation. The lower panels of Fig. 2a–d show schematics of the samples, in which the geometries of the topological TI–OI interfaces are denoted by the yellow dashed lines. Two different defects, including a cavity with randomly filled holes (Fig. 2b) and a randomly disordered lattice of perforated holes (Fig. 2c) are placed in the original interface (Fig. 2a). In addition, a *Z*-shaped interface with two 120° sharp bends (Fig. 2d) is also fabrication for test. Note that in all these configurations, spin-mixing mechanisms (effective magnetic impurities) are absent. Elastic energy distributions obtained in simulations and measurements are shown in the upper panels of Fig. 2a–d with the measured transmission spectra shown in Fig. 2e, confirming the robustness and flexibility of this elastic waveguiding scheme, which is characterized by the elastic waves detouring around all these arbitrary defects and bends while maintaining nearly a loss-free elastic transmission. In comparison, for traditional elastic waveguides formed by OI-OI interface, the same defects cause distinct elastic resonances and the bends severely inhibit the elastic forward transmission. As a result, a decreased transmission or even a total reflection can be observed due to strong elastic backscattering (see Supplementary Note 4). It should be noticed that the robustness in our system against bends is valid for any bending angle besides the one, 120°, shown in our experiments.

Remarkably, since its structural simplicity and (electro-mechanical) transducing accessibility, our elastic waveguide is thoroughly scalable and CMOS/MEMS compatible (see discussion in Supplementary Note 4). It can be exploited as a standard component/platform with great versatility for future large-scale planner integration of phononic devices in, e.g., piezo-(opto-)mechanical systems^38–40^ operating over GHz and even beyond.

### Monolithic elastic topological whisper-gallery resonator

**Fig. 3:**
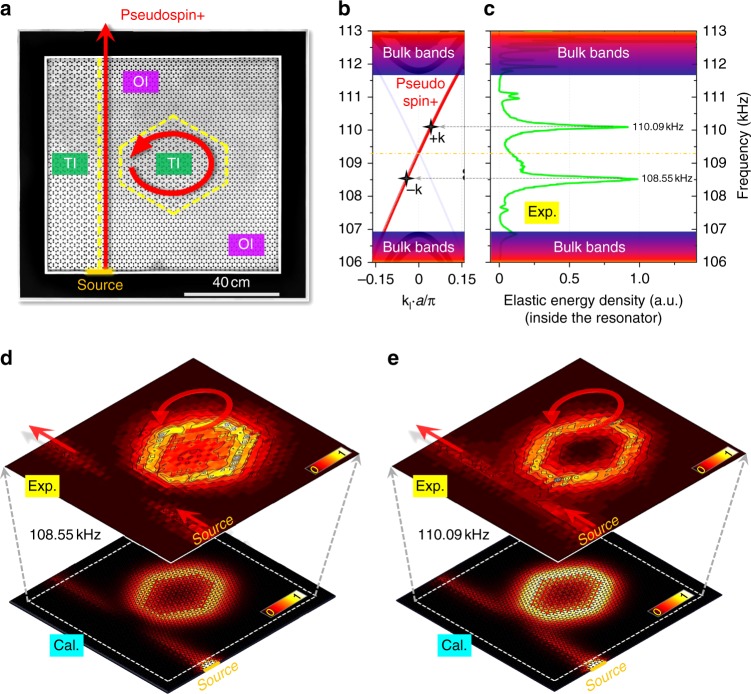
Elastic topological whisper-gallery resonator. **a** Photograph of a topologically protected waveguide-coupled elastic resonator, consisting of one OI and two TIs. Two TI–OI interfaces exist as indicated by yellow dashed lines: one corresponding to a two-port straight waveguide on the left, the other corresponding to a hexagon-shaped waveguide on the right, i.e., a topological whisper-gallery resonator in an unconventional geometry. **b** Calculated dispersion of the elastic helical edge states, pseudospin+ is actually excited when we placed an ultrasonic transducer at the bottom of the straight waveguide. **c** Experimentally measured spectrum of elastic energy density inside the whisper-gallery resonator, having two distinct peaks at the frequencies of 108.55 kHz (at *−***k**) and 110.09 kHz (at +**k**), respect to the Dirac frequency (at **k** = 0) of the helical edge states. The load Q-factor for the resonator modes at these two frequencies are remarkable exceeding 10^3^. **d,** **e** Calculated and experimentally imaged elastic energy-density distributions for these two resonances, showing highly localized energy distribution inside the resonator

The above novel waveguiding scheme for the elastic waves can be leveraged to create counter-intuitive devices that have immediate applications. An example of significance is a monolithically integrated elastic topological whisper-gallery (WG) resonator. WG resonators have found key applications and have become an integral part in many fields such as coherent energy storage and source generation. However, the circulating WG modes in conventional resonators lose their energy gradually, owning to scattering losses from surface imperfection and geometric variation along the circulating path. Many efforts have been devoted to reduce scattering losses by developing advanced material processing and fabrication techniques^41–43^. Here the topological design offers a completely novel and probably the most advantageous alternative for combating the scattering losses. For illustration, two TI–OI interfaces are constructed as shown in Fig. 3a: one straight waveguide of two-ports on the left, and one closed hexagon-shaped waveguide on the right, i.e., the WG resonator with six abrupt corners. Our WG resonator has a much reduced rotation symmetry compared with traditional ring or disk resonators, thus completely against common wisdom for high-performance resonator design. It is, however, very efficient and practically viable thanks to the topological protection. Specifically, the spin-momentum locking restricts the pseudospin+, excited by the bottom transducer, to only two spin-preserving scattering channels as no spin-flipping scatters presented in our case. These two scattering channels are the upward channel in the straight waveguide and the counter-clockwise circulating channel in the hexagonal resonator, respectively.

The spectrum for the elastic energy density inside the hexagonal resonator is measured and shown in Fig. 3c. Two strong peaks exist, suggesting the two scattering channels can efficiently couple with each other via multiple scattering processes in the vicinity between the straight waveguide and the WG resonator. Their resonance frequencies are 108.55 kHz and 110.09 kHz, respectively. By examining the calculated dispersions of our elastic helical edge states (shown in Fig. 3b, symmetric to the center of the Brillouin zone), it is found that these two frequencies are almost symmetric about the Dirac frequency (109.34 kHz). Thanks to the helical edge states’ linear dispersion near the Dirac frequency, the Bloch momentums corresponding to these two frequencies have the same magnitude but opposite direction. Since the self-consistence condition imposed by the WG resonance involves only the magnitude of the Bloch momentum, the resonance always emerges as a pair around the Dirac frequency, which is very unique to topological systems.

These two resonances are further examined in real space by imaging the field of this device. The obtained normalized elastic energy distribution at these two resonance frequencies, shown in Fig. 3d, e indicates that the elastic energy is mostly localized inside the hexagonal resonator. The loaded Q-factor for the resonance at 110.09 kHz is measured to be more than 1200 (with a 3 dB bandwidth of 0.09 kHz and a mode volume of ~500 cm^3^), a remarkable value for acoustic devices at this large scale when compared with that (usually about 500–1000) of a high-quality tuning fork or a commercial miniaturized SAW resonator^44^. Considering the fact that our prototype device has not gone through any rigorous optimization, even higher Q-factor is very likely by further exploiting other substrates or geometrical configurations. Our study here clearly exemplifies the advantage that is readily obtained in practice from the topological robustness of the edge states. It thus could be applied straightforwardly into a broad range of relevant fields, where coherence enhancement is in great need, e.g., high-sensitive shear force detection, elastic energy harvesting^45^, optomechanical sensing^46^, and coherent phononic/photonic laser sources^47^, etc.

### Elastic pure pseudospin current at TI boundary

**Fig. 4:**
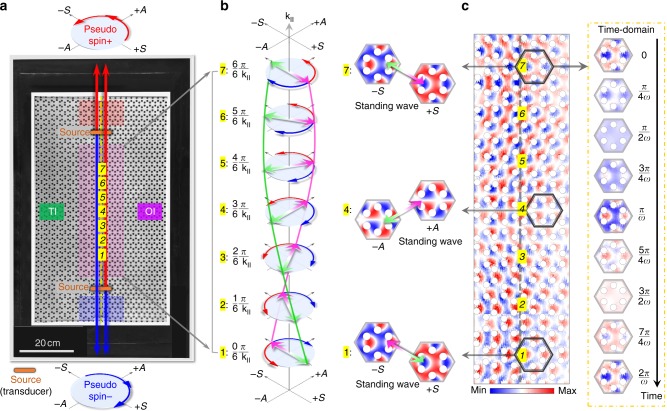
Pure pseudospin current of elastic waves. **a** Experimental configuration for investigating pure pseudospin current of elastic helical edge states. Two (orange) identical elastic sources (ultrasonic transducers) working at same power and frequency (111 kHz) are placed in two different places of a straight TI–OI interface, dividing the interface into three regions (top, middle, and bottom). In the top (red) region, there only exists upward-traveling pseudospin+ , superimposed from two individual pseudospins+ , each originated from one of the two sources. Similar situation in the bottom (blue) region, instead there only exists downward-traveling pseudospin−. Remarkably, pseudospin+ and pseudospin− coexist in the middle (green) region. **b** Physical image of the pseudospin current can be well exhibited by the vector sum of the pseudospin+ and the pseudospin− in their (*S*/*A*) spin space at time-domain. Because the anti-clockwise pseudospin+ (red arrows, *S* + i*A*) and the clockwise pseudospin− (blue arrows, *S* − i*A*) are with the same amplitude and velocity (that is, rotation speed in spin space), thus in any location along the TI–OI interface, like seven representative locations from #1 to #7, the vector sum of these two elastic pseudospins will inevitably presents a single polarization feature in the spin space all over time, e.g., ∓*S* ↔ ±*S* (position #1 and #7) or +*A* ↔ −*A* (position #4) as illustrated by the (violet-green) double-headed arrows. Meanwhile, along the interface (k_II_), the vector sum of these two elastic pseudospins (that is, the single polarization in spin space) will evolve in a one-way spiral fashion, shown as the (violet-green) double helix, owning to the spin-momentum locking. **c** Experimentally measured elastic field distribution of the out-of-plane displacement along the TI–OI interface conforming the pure pseudospin current. Particularly, at the three representative locations marked by black hexagons, the elastic displacements verify single polarization normal modes −*S* ↔ +*S* (position #1), +*A* ↔ −*A* (position #4) and +*S* ↔ −*S* (position #7), all only oscillates harmonically with time as shown in the right-most panel. This is a clear indication of the formation of a standing-wave with one-way spiral in the real space

As the signature of the QSHE, pure spin current with zero charge current (*j*_c_ = 0, *j*_s_ ≠ 0) can be imaged and studied using a configuration shown in Fig. 4a. It contains a straight topologically protected waveguide with two identical transducers placed face to face on the waveguide, each of which will excite both an upward traveling pseudospin+ and a downward traveling pseudospin− at the same frequency simultaneously. In the top region (marked by red), there only exists an upward-traveling pseudospin+ , superimposed from two individual pseudospin + , each originated from one of the two transducers. This fact is precisely observed and confirmed in experiment (see time-harmonic Supplementary Movie 1). Similar results can be obtained in the bottom region (marked by blue), where only downward traveling pseudospin− exists (see Supplementary Movie 2).

Remarkably in the region (marked by purple) between the two transducers, there exist simultaneously an upward traveling pseudospin+ excited by the bottom transducer and a downward traveling pseudospin− excited by the top transducer. Pure pseudospin current (without energy flow) in this region is thus determined by the coherent superposition of these two pseudospins, as illustrated in Fig. 4b. When the pseudospins exited by two transducers have the same amplitude, they (in red and blue arrows) are effectively annihilated anywhere in this region, due to their same rotating phase and group velocity but opposite direction. Consequently, this process leaves behind only a non-spinning or an equivalent linear vector field component in the *S*/*A* normal mode basis. Since the phase difference between the two pseudospins varies linearly with location (e.g., from position#1 to position#7), the resulted vector direction of the linear component rotates correspondingly in a linear fashion to form a well-defined spatial chirality, as illustrated by the DNA-like double helix marked by the violet and green traces. Note that in this case the helix is left-handed at the right boundary of TI, and a right-handed chirality can also be obtained but at the left boundary of TI.

In experiment, the out-of-plane displacement distribution in this region is obtained at a deep subwavelength resolution and shown in Fig. 4c (see Supplementary Note 6 for complete field information, and Supplementary Movie 3 for its temporal evolution). At any location in this region, e.g., the top position #7, the field pattern in a unit cell remains the same, but its intensity blinks with time, as illustrated in the right panel. This indicates the formation of a standing wave pattern. Along the waveguide in this region, the symmetry of the field in the unit cell undergoes periodic changes, e.g., symmetric—antisymmetric—symmetric or equivalently $\pm {\it{S}}\cdot\cdot\cdot \mp {\it{A}}\cdot\cdot\cdot \mp {\it{S}}$. Consequently, the standing-wave pattern is seen to spiral in one-way fashion, representing the signature of pure pseudospin current, i.e., no energy flow but a pseudo angular momentum transport in real space.

Up to date, a satisfactory visualization of the edge states of the QSHE, especially the corresponding spin dynamics in spatial and temporal space, remains a challenge in electronic systems, because of the limitations set by the extreme scale of electron waves and difficulties lying in material growth and device fabrication. Our observation in elastic systems provides a comprehensive scenario to the fundamental role of the spin/pseudospin dynamics in topological states, opening up a possibility towards potential bosonic pseudo-spintronics with great flexibility, stability and signal fidelity.

## Discussion

We realized and confirmed with abundant visual data an elastic topological material in a continuous solid-state medium. Using this new material, we have captured convincing real space evidences for elastic helical edge states, pseudospin dynamics and other related novel phenomena with a never-before deep sub-wavelength resolution. All our findings obtained here may be directly leveraged to investigate and ultimately pave the way for future phonon-based high performance (in terms of energy efficiency, information capacity, and signal integrity) information processing devices on a variety of commonly used material systems, e.g., Si^48^, AlN^49^, LiNbO_3_^50^, and on many emerging molecular or two-dimensional piezoelectric materials (like MoSe_2_, WTe_2_ in the transition metal chalcogenide families^51^). This material may also reinforce the recent developed study of quantum acoustics, since a precise controlled elastic phonon may interact with various quantum systems, e.g., superconducting qubits coupled via surface^52^ and bulk^53^ acoustic waves. To further demonstrate the capability of this new material, some unprecedented and high-performance elastic devices such as an elastic topologically protected whisper-gallery resonator are constructed and studied thoroughly in real-space, potentially impacting a broad range of relevant fields. Last, in the view point of fundamental sciences, our findings may even underpin the foundations for understanding the topological properties and spin related transport behaviors in other bosonic and fermionic materials under the theory of great unity of waves.

## Methods

### Sample preparation

Our samples are prepared exclusively on polished stainless-steel plates (Type 201, mass density 7903 kg m^−3^) with a fixed plate thickness 7.82 mm. Their elastic parameters are determined by ultrasonic scattering echo method, i.e., 201.075 GPa for Young’s modulus and 0.3254 for Poison ratio. The plates are perforated on a precision CNC milling machine to create phononic crystals with identical hole radius *r* = 3.52 mm and lattice constant *a*_0_ = 11.29 mm. An impedance matched sound proof adhesive (epoxy resin and tungsten powder) is coated on the circumference of the sample to prevent unwanted back reflection.

### Numerical calculation

Elastic band structure calculations, eigen mode distributions and surface acoustic energy distributions shown in this work are conducted by a three-dimensional finite element method using acoustic module of commercial software COMSOL MULTIPHYSICS.

### Experimental apparatus

Broadband piezoelectric ultrasonic transducers (center frequency at 110 kHz) are attached to the sample surface as an excitation source. They are driven by a function generator followed by a power amplifier. A fiber laser based two-wave mixing interferometer with a lock-in amplifier is used to interrogate the amplitude and the phase information of the back reflected optical measurement beam from the sample surface. The field profile of the elastic wave (SAW) is then mapped out in a point by point fashion based on the information of the reflected optical measurement beam. The radiating pressure exerted by our measurement optical beam on the plate surface is estimated to be negligible to the shear-force pressure caused by the SAWs. Thus, the SAW propagation is unaffected by our optical beam. In addition, the imaging resolution is determined primarily by the size of our optical beam, which can be focused down to less than 1/50 of the SAW wavelength. All these advantages combined enables us to present the first non-disturbing deep subwavelength imaging of topological edge states and helical pseudospin transport.

### Data availability

The datasets within the article and supplementary information in the current study are available from the authors upon request.

## Electronic supplementary material

Supplementary Information

Description of Additional Supplementary Files

Supplementary Movie 1

Supplementary Movie 2

Supplementary Movie 3
