# Author Correction: Elastic pseudospin transport for integratable topological phononic circuits

**DOI:** 10.1038/s41467-019-08589-0

**Published:** 2019-01-31

**Authors:** Si-Yuan Yu, Cheng He, Zhen Wang, Fu-Kang Liu, Xiao-Chen Sun, Zheng Li, Hai-Zhou Lu, Ming-Hui Lu, Xiao-Ping Liu, Yan-Feng Chen

**Affiliations:** 1 National Laboratory of Solid State Microstructures & Department of Materials Science and Engineering, Nanjing University, Nanjing 210093, China; 2 Collaborative Innovation Center of Advanced Microstructures, Nanjing University, Nanjing 210093, China; 3 Jiangsu Key Laboratory of Artificial Functional Materials, Nanjing 210093, China; 4 Shenzhen Institute for Quantum Science and Engineering and Department of Physics, South University of Science and Technology of China, Shenzhen 518055, China

Correction to: *Nature Communications*; 10.1038/s41467-018-05461-5, published online 06 August 2018

The original version of this Article contained errors in the second sentence in the legend of Fig. 1, which incorrectly read ‘These two elastic insulators are identical in lattice constant *a* (3*a*_0_), plate thickness (0.4*a*_0_), and radius of perforated holes *r* (0.18*a*_0_) but different hole-center distance characterized by *b*.’ The correct version states ‘plate thickness ($\sqrt 3$ × 0.4*a*_0_)’ in place of ‘plate thickness (0.4*a*_0_)’ and ‘radius of perforated holes *r* ($\sqrt 3$ × 0.18*a*_0_)’ rather than ‘radius of perforated holes *r* (0.18*a*_0_)’.

The first sentence of the ‘Sample preparation’ section of the Methods originally incorrectly read ‘Our samples are prepared exclusively on polished stainless-steel plates (Type 201, mass density 7803 kg m^−3^) with a fixed plate thickness 7.82 mm.’ In the corrected version, ‘mass density 7903 kg m^−3^’ replaces ‘mass density 7803 kg m^−3^’.

The second sentence in the legend of Supplementary Fig. 3, originally incorrectly read ‘The symmetry of the phononic crystal remains unchanged as C_6*ν*_, and thickness of the substrates *H* (equals to 0.4*a*_0_), lattice constant a (equals to 3*a*_0_) and radius of perforated holes *r* (equals to 0.18 *a*_0_) maintain constant.’ The correct version states ‘√3 × 0.4*a*_0_’ in place of ‘0.4*a*_0_’ and ‘√3 × 0.18*a*_0_’ rather than ‘0.18*a*_0_’.

This has been corrected in both the PDF and HTML versions of the Article.

