# Supplementary material for: Elastic pseudospin transport for integratable topological phononic circuits
Source: Nat Commun. 2018 Aug 6;9:3072. doi: 10.1038/s41467-018-05461-5 (PMC6078995; doi:10.1038/s41467-018-05461-5)
Supplement: Supplementary file 1 — Supplementary Information [file 41467_2018_5461_MOESM1_ESM.pdf]

Supplementary Information:

Elastic Pseudospin Transport for Integratable Topological Phononic Circuits

Si-Yuan Yu[1,2], Cheng He[1,2], Zhen Wang[1], Fu-Kang Liu[1], Xiao-Chen Sun[1], Zheng Li[1], Hai-Zhou Lu[3], Ming-Hui Lu[1,2], Xiao-Ping Liu[1,2], and Yan-Feng Chen[1,2]

1. National Laboratory of Solid State Microstructures & Department of Materials Science and Engineering, Nanjing University, Nanjing, 210093, China

2. Collaborative Innovation Center of Advanced Microstructures, Nanjing University, Nanjing, 210093, China

3. Institute for Quantum Science and Engineering and Department of Physics, South University of Science and Technology of China, Shenzhen 518055, China

Supplementary Note 1:

Elastic accidental four-fold degeneracy for mode inversion with an overlapped bandgap

Band folding[1] is a convenient method for obtaining four-fold degeneracy at the Brillouin zone centre. However, band folding requires a clean Dirac point at the K/K' point of a triangular/honeycomb lattice where there are no other modes besides those that form the conical Dirac dispersions. This requirement can be achieved in some situations, e.g., in fluid/air-borne phononics, in microwave photonics ("Crystalline metamaterials for topological properties at subwavelength scales", *Nat. Commun.* 8, 16023 (2017) and in "Visualization of unidirectional optical waveguide using topological photonic crystals made of dielectric material" (arXiv:1610.07780). This is not, however, always the case in many other configurations.

We want to emphasize a very essential point that makes our work stand out: Our work is geared towards artificial acoustic materials that can be practically scaled for integrated applications. In solid-state elastic systems, compared with using subwavelength meta-structures[2] or complex structures that include composite materials[3], a lattice of uniform perforated holes (at wavelength scales) in a plain substrate is the most concise and attainable configuration for practical preparations. The footprint can be maximally scaled down, with micro/nano processing technology that is currently used in CMOS/MEMS manufacturing[4], for chip-scale enabling integrated devices with much higher working frequencies to target key RF applications. Unfortunately, within this concise configuration, there is no way to find clean Dirac points at the K/K' points, and thus, four-fold degeneracy cannot be achieved by band folding.

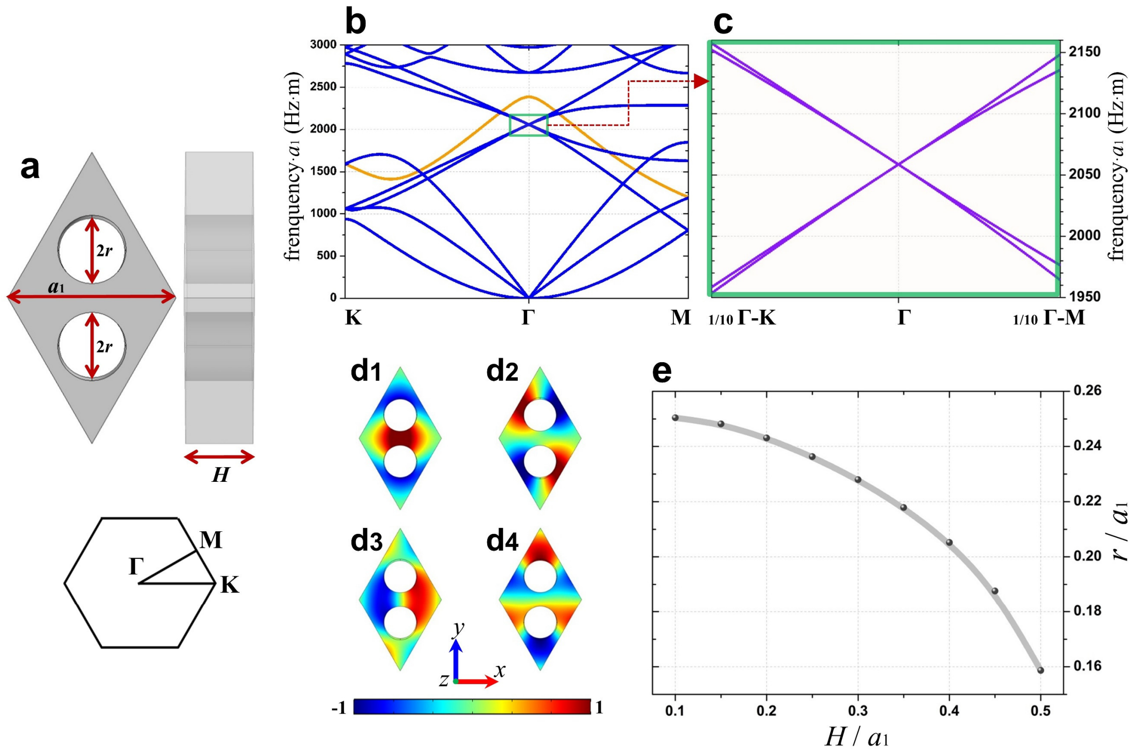

Supplementary Figure 1 | Elastic accidental four-fold degeneracy in a perforated phononic crystal plate in a honeycomb lattice. (**a**) Geometric configuration of a unit-cell. There are only two adjustable geometric parameters, H (plate thickness) and r (radius of the holes), ignoring the normalized lattice constant, a_1. By precisely adjusting the two parameters, one can achieve an elastic four-fold accidental degeneracy in the centre of the Brillouin zone, as shown in the band diagrams (**b**) (global) and (**c**) (zoomed-in). (**d**) Elastic displacement distributions in the (out-of-plane) z-direction of the four degenerate modes. Yellow dispersions consist of shear horizontal modes that are intrinsically different from the other modes shown in the band structure. (**e**) For a certain a_1, this accidental degeneracy can only be achieved by a monotonic H-r relationship, *i.e.*, the thicker the plate, the smaller the radius of the holes.

As shown in **Supplementary Fig. 1**, in a simple honeycomb lattice of our perforated phononic crystal plate, four-fold degeneracy is accidentally achieved at the Brillouin zone centre by simply adjusting the only two geometric parameters, H (plate thickness) and r (radius of the holes), and ignoring the normalized lattice constant, a_1. Note that the H-r relationship that achieves this degeneracy is monotonic.

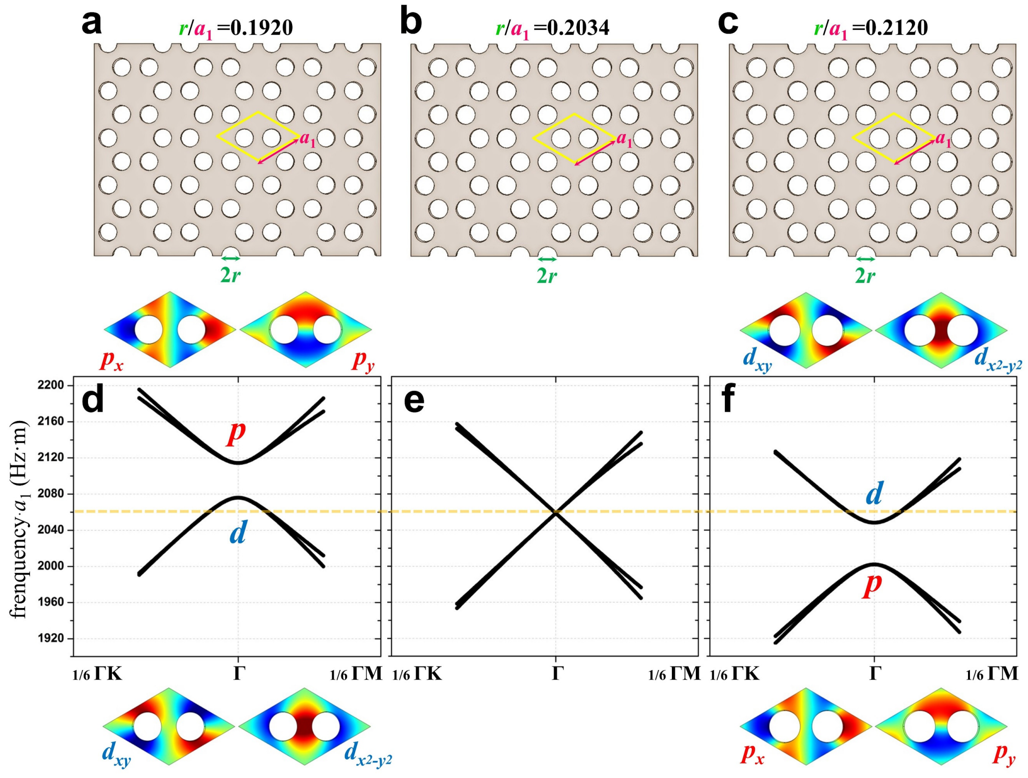

Supplementary Figure 2 | Elastic band inversion via geometrical revolution in a honeycomb lattice. Panels (**a**) through (**c**): Geometric evolution of the phononic crystal plate. The C_{6v} (honeycomb lattice) symmetry of the phononic crystal remains unchanged, and the thickness of the plate, H, maintains at a definite value of H=0.4a_1. The only variation is the radius of each of the identical perforated holes, r, which increases from (**a**) 0.1920a_1 to (**b**) 0.2034a_1 and then to (**c**) 0.2120a_1. Panels (**d**) through (**f**): Band diagrams near the Brillouin zone centres corresponding to the above three cases, which illustrate an elastic band inversion. When r is equal to (**a**) 0.1920a_1 (<0.2034a_1), the phononic crystal has two two-fold degenerate states, denoted as p_x/p_y and $d_{x2\text{-}y2}/d_{xy}$, separated by an elastic bandgap. When r increases to (**b**) 0.2034a_1, the bandgap disappears, and an accidental four-fold degeneracy is formed. When r further increases to (**c**) 0.2120a_1, the band gap reappears along with two inverted two-fold degenerate states, $d_{x2\text{-}y2}/d_{xy}$ and p_x/p_y. The surrounding insets are the elastic (out-of-plane) displacement distributions of the degenerate states.

In this simple configuration, if H is fixed, band inversion can also be achieved by adjusting r (as shown in **Supplementary Fig. 2),** and vice versa, for a fixed r. Unfortunately, however, in this simple configuration, elastic bandgaps within the inverted bands cannot be achieved at the same frequencies (Supplementary Fig. 2d vs. Supplementary Fig. 2f), preventing the construction of topologically protected (non-radiative) edge states. Consequently, the implementation of a super cell

(three times larger than the original unit cell in the honeycomb lattice) in our main text (Fig. 1) is not to take advantage of band folding, although they look similar at first glance.

Instead, the implementation of a super cell is used to set a new degree of freedom in the geometric parameter (in addition to H and r) to modulate the band structure and to achieve bandgaps with topological inversions at the same frequency range without violating the C_{6v} symmetry.

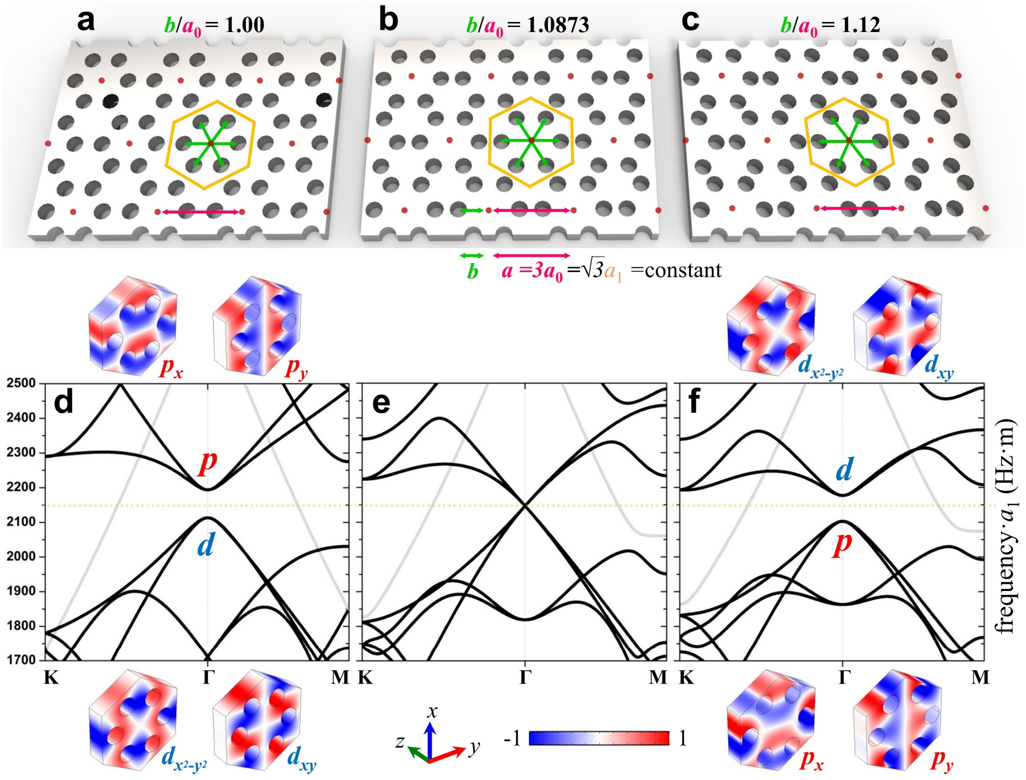

Supplementary Figure 3 | Elastic band inversion with an overlapped bandgap in a larger unit-cell. Panels (**a**) through (**c**): Geometrical revolution of the phononic crystal plate composed of identical perforated holes. The symmetry of the phononic crystal remains unchanged since the C_{6v} lattice constant, a (a=3a_0= √ 3a_1), the thickness of the substrates, H (0.4a_1), and the radius of the perforated holes r (0.18a_1) remain constant. However, in each unit-cell, as the yellow hexagons indicate, the length between the centre of the six perforated holes and the centre of the unit-cell, b, increases from (**a**) 1.00a_0, to (**b**) 1.0873a_0, and (**c**) 1.12a_0. Panels (**d**) through (**f**): Band diagrams corresponding to the three cases illustrating a band inversion with an overlapped bandgap. In the original lattice (**a**) b=1.000a_0, two two-fold degenerate states, which can be denoted as p_x/p_y and $d_{x2\text{-}y2}/d_{xy}$, are separated by an elastic bandgap (ignoring the grey shear-horizontal modes). When b increases to a unique value, e.g., (**b**) 1.0873a_0, the band-gap disappears and an accidental double Dirac cone with a four-fold degeneracy is formed. When b

further increases, e.g., to (**c**) $1.12a_0$, the band-gap reappears along with two inverted two-fold degeneracies corresponding to d_{x2-y2}/d_{xy} and p_x/p_y. The insets surrounding the band diagrams are the elastic displacement distributions of the degenerate elastic eigenstates in the (out-of-plane) z direction.

As demonstrated in **Supplementary Fig. 3**, in the three times larger unit-cell (yellow hexagonal regions), there are six identical perforated holes, and the desired new adjustable geometric parameter, *b*, is created and is measured from the centre of the perforated holes to the centre of hexagonal unit-cell. The tuning procedure gradually changes to *b*, while the lattice constant, *a* ($a=3a_0=\sqrt{3}a_1$); thickness of the substrates, *H* ($0.4a_1$); and the radius of the perforated holes, *r* (equals to $0.18a_1$), remain constant. Of particular note is the fact that *r* is now even smaller than $0.1920a_1$, which is the value used in Supplementary Fig. 2a. Ignoring the grey shear-horizontal modes, when *b* is relatively small, for example, when $b=a_0$ (Supplementary Fig. 3a, a standard honeycomb lattice in this case), there is an elastic band-gap. Two pairs of two-fold degenerate surface acoustic states exist at the Brillouin zone centre corresponding to p_x/p_y and d_{x2-y2}/d_{xy}. When *b* is increased to nearly $1.0873a_0$ (Supplementary Fig. 3b), the elastic bandgap vanishes, resulting in an accidental double Dirac point with four-fold degeneracy. A further increase in *b*, *e.g.*, to $b=1.12a_0$ (Supplementary Fig. 3c), results in an elastic band inversion that is characterized by the flipping of the p and d modes. Finally, two elastic insulators with band inversions have an overlapped bandgap.

Increasing the hole-centre distance, *b*, an inversion of the *p/d* modes occurs along with a topological phase transition of the bulk bands (with the spin Chern number changing from zero to non-zero). In fact, theoretically, based on the BHZ model of the quantum spin Hall effect (QSHE) in a two-dimensional electron topological insulator, the Hamiltonian of our system can be written as[1]:

$$\mathcal{H}(k)=\begin{pmatrix} -M-\frac{(D+F+2N)}{2}k^2 & Ak_+ & 0 & 0 \\ A^*k_- & M+\frac{(D+F+2N)}{2}k^2 & 0 & 0 \\ 0 & 0 & -M-\frac{(D+F+2N)}{2}k^2 & Ak_- \\ 0 & 0 & A^*k_+ & M+\frac{(D+F+2N)}{2}k^2 \end{pmatrix} \quad (1)$$

According to the above equation, an inversion of the modes between the bulk gap can be achieved by an independent variation of the parameter *M*, which describes the energy difference between the *p* (-like) and *d* (-like) modes.

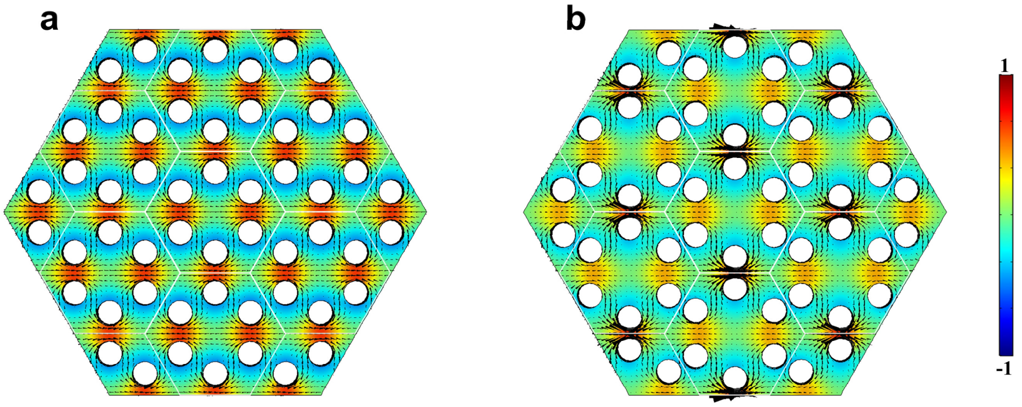

Supplementary Figure 4 | d_{x2-y2} -like mode of different hole-centre distance. Colour maps represent for out-of-plane displacement fields of the d_{x2-y2} -like bulk mode in a super cell of nine unit-cells. Black arrows indicate the densities of elastic energy flux. As we increase the hole-centre distance from (**a**) $b=a_0$ to (**b**) $b=1.12a_0$, the characteristics of the elastic coupling from one unit-cell to its near-by neighbours distinctly changes, which manifests as the concentration of the elastic energy at particular areas on the boundaries of each unit-cell.

Specific to our solid-state elastic model, as the hole-centre distance varies, the characteristics of the elastic coupling from one unit-cell to its near-by neighbours distinctly changes. As shown in **Supplementary Fig. 4**, in the original honeycomb lattice (hole-centre distance $b=a_0$), the elastic energy fluxes between neighbouring unit-cells are evenly spread around every corner and boundary of every hexagonal unit-cell. On the contrary, in a lattice with a larger hole-centre distance ($b=1.12a_0$), the elastic energy fluxes are now concentrated in particular areas, *i.e.*, between the nearest two perforated holes from two different unit-cells in the vertical direction. Since M in the

theoretical model is closely related to the elastic coupling between neighbouring unit cells, a refined conclusion can be drawn: "varying the hole-centre distance" changes the characteristics of the coupling between neighbouring unit cells (in the theoretical model that describes our system), which leads to the "inversion of the modes".

In bosonic systems, the key factor for realizing the QSHE is to construct artificial bosonic spin-½ states (herein referred as pseudospins) under a pseudo (fermi-like) TR symmetry ($T_p^2=-1$). Generally, these pseudospins can be emulated through polarization or modal hybridization. For instance, two degenerate modes, M_1 and M_2, can be hybridized to construct these pseudospins: spin+/− ≡M_1+iM_2/M_1–iM_2, only if $M_1 \xrightarrow{T_p} M_2$ while $M_2 \xrightarrow{T_p} -M_1$.

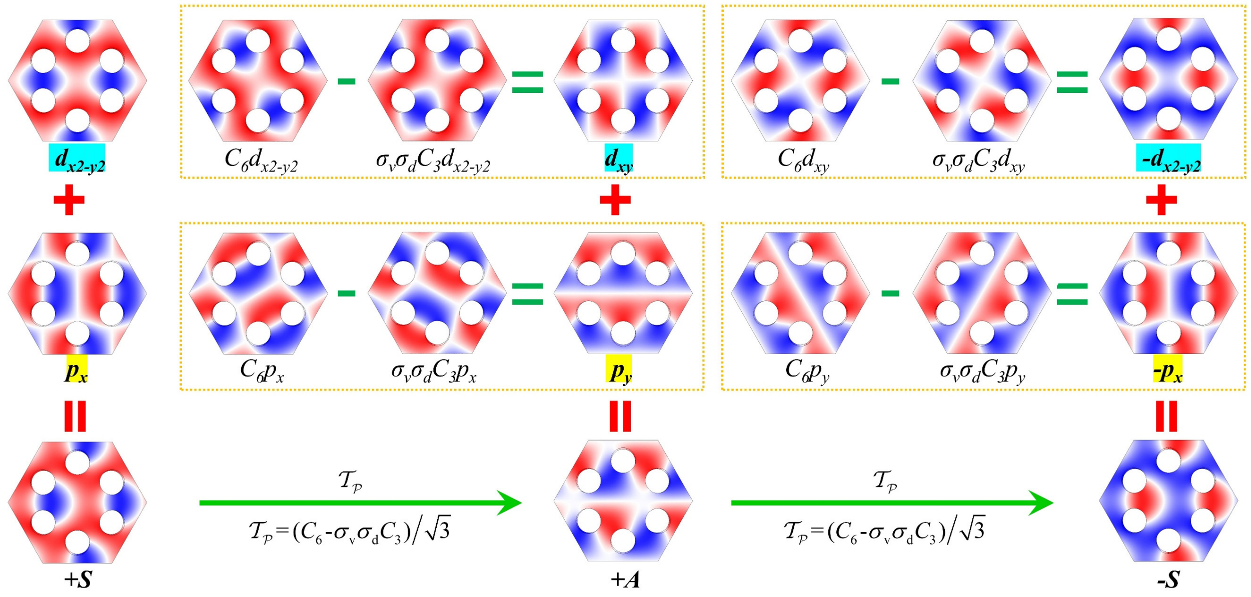

Supplementary Figure 5 | Evolution of the p and d modes (and corresponding S and A) by the pseudo time-reversal operator, $T_p=(C_6-\sigma_v \cdot \sigma_d \cdot C_3)/\sqrt{3}$

As demonstrated in **Supplementary Fig. 5**, bulk p and d modes hybridize to form a pair of normal modes, *i.e.*, one symmetric mode, $S=(p_x+d_{x2-y2})/\sqrt{2}$, and one anti-symmetric mode, $A=(p_y+d_{xy})/\sqrt{2}$. Subsequently, these two normal modes are used as a basis to construct the

required two pseudospins, S+iA and S−iA, which are protected by the pseudo TR symmetry (T_p^2=−1), as visually validated by placing the T_p operator on the S/A basis, *i.e.*, $+S \xrightarrow{T_p} +A \xrightarrow{T_p} -S \xrightarrow{T_p} \cdots$. Specific to our system, this T_p is equal to $(C_6 - \sigma_v \cdot \sigma_d \cdot C_3)/\sqrt{3}$.

To form non-radiative boundary transmission modes, it is first necessary to have two "insulators" with their forbidden bands energetically aligned. When we attach these two "insulators" side-by-side to form a boundary, boundary modes will appear. Some of the boundary modes will exist within the frequency range of the forbidden bands, and thus, they are unable to be converted to any bulk modes and radiate outside the boundary. These non-radiative boundary modes can be topologically protected or not depending on the topological properties of the energy bands of the two "insulators" on both sides.

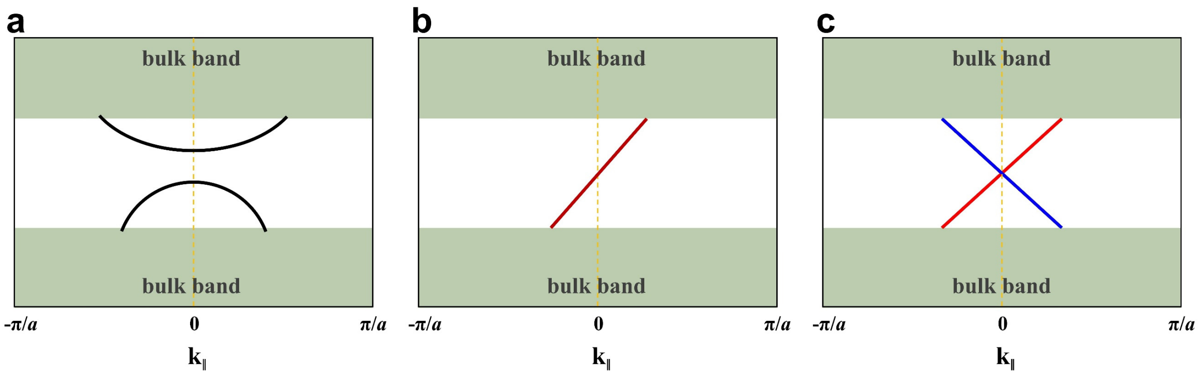

Supplementary Figure 6 | Several kinds of edge states. (**a**) Topological trivial edge states. Each dispersion is symmetric about k=0, and there is always a band-gap of the edge state. Panels (**b**) and (**c**): Topological non-trivial edge states in QHEs and QSHEs. Each dispersion is gapless and touches both the lower and upper bulk bands.

If the properties of the energy bands of the two "insulators" are topologically the same, which can be characterized by topological invariants, such as the Chern number (C) or the spin Chern number (C_S), there will be only trivial edge states. The dispersions of the trivial edge states are similar to those shown in **Supplementary Fig. 6a**. Every energy dispersion is symmetric about k=0, and thus, there are always two modes at +k and −k at the same frequency. The backscattering of the

boundary modes (Mode $_{+k}$ ↔ Mode $_{-k}$) is unavoidable.

On the contrary, if the properties of the energy bands of the two "insulators" are topologically different (for instance, with an inversion of the modes), the dispersions of the edge states will be as shown in Supplementary Fig. 6b and Supplementary Fig. 6c, i.e., each dispersion needs to be "gapless", as shown in **Supplementary Fig. 7**, much like the chiral edge states in QHEs or the helical edge states in QSHEs. Each dispersion is no longer symmetric about k=0, instead, there is only one mode for each wave-vector (k) at any frequency in the bulk gap making the back-scattering of the boundary modes (Mode $_{+k}$ ↔ Mode $_{-k}$) impossible.

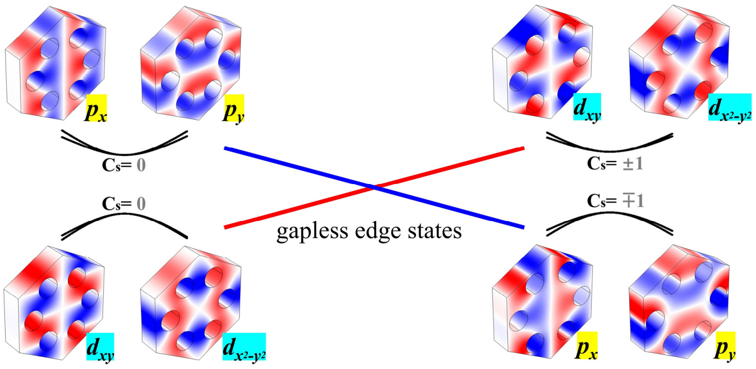

Supplementary Figure 7 | Band inversion and the corresponding gapless edge states: (left) Topological trivial "insulator" with *p* and *d* (-like) states for the upper and lower bands between the forbidden bands; (right) topological non-trivial "insulator" with the *p* and *d* (-like) states reversed. If these two insulators are attached to form a boundary, the edge states will be "gapless" between the *p* and *d* (-like) bulk modes.

In our system, analogous to the quantum spin Hall effect with elastic pseudospins, the topological invariant is the spin Chern number (C_s). According to our calculations, the C_s value in one insulator is different by one from the insulator on the other side of the boundary. Specifically, the spin Chern number is zero in one insulator (as shown in the left panel of Supplementary Fig. 7) and is nonzero in the other (as shown in the right panel, C_s is ±1 for the upper two bands above the forbidden band and ∓1 for the lower two bands). Hence, the dispersions of the boundary modes must be "gapless" according to the "bulk-boundary correspondence", as the calculated results show in our main text.

In general, topological phase transitions of the energy bands can be realized through the

following means: 1) Find a topological phase transition point of the energy bands, *i.e.*, a Dirac degeneracy, at which the material is "semimetal"; 2) open the Dirac degeneracy in two opposite ways and thus form two "insulators" with inverted bulk modes. Take our sample as an example; the topological phase transition point is the four-fold Dirac degeneracy, as shown in Supplementary Fig. 3e. As we deform the lattice by decreasing the hole-centre distance, the Dirac degeneracy will be lifted to form an "insulator" with *p* (-like) modes at higher frequencies and *d* (-like) modes at lower frequencies, as shown in Supplementary Fig. 3d. On the contrary, as the hole-centre distance increases, an "insulator" with inverted *p* and *d* (-like) modes will be formed, as shown in Supplementary Fig. 3f.

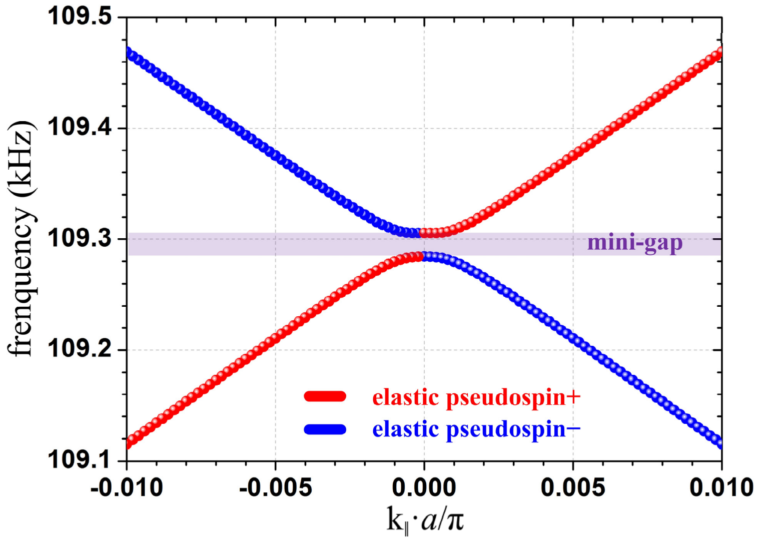

Supplementary Figure 8 | A mini-gap of the two elastic helical edge states at $k_{\parallel}$=0.

The zoomed-in calculated band diagram of Fig. 1b in the main text shows that there is a very small bandgap between the two elastic helical edge states at $k_{\parallel}$=0, as shown in **Supplementary Fig. 8**. This mini-gap is caused by the breaking of the C_{6v} symmetry at the interface between the OI (b=1.00a_0) and TI (b=1.12a_0). However, the mini-gap here is only approximately 0.01 kHz according to the calculation, which is much smaller than (1/500) the bulk band-gap (approximately 5 kHz, from approximately 106.7 kHz to 111.7 kHz). Consequently, it is too small to be experimentally detected throughout our work.

Supplementary Note 2:

Calculated Berry curvature and spin Chern numbers

To numerically calculate the spin Chern number, we start from the general case of the Chern number in the discretized Brillouin zone (BZ)[5]. The basic equation can be read as

$$c_n = \frac{1}{2\pi i}\int d^2 k F_{xy}(k) \tag{2}$$

where $F_{xy}(k) = \partial_x A_y(k) - \partial_y A_x(k)$ represents the Berry curvature. The Berry connection $A_\mu(k)$ ($\mu = x, y$) can be described as

$$A_\mu(k) = \langle n(k)|\partial_\mu|n(k)\rangle \tag{3}$$

where $|n(k)\rangle$ is the wave function of the *n*th Bloch band.

In the two-dimensional discrete BZ, we can change the differentiation to the logarithm and summation as follows,

$$A_\mu(k) \to \frac{\ln U_\mu(k_l)}{\Delta_\mu} \tag{4}$$

where $U_\mu(k_l) = \langle n(k_l)|n(k_l + \Delta_\mu)\rangle$. Subsequently, the Berry curvature and corresponding Chern number can be described as

$$\begin{aligned} F_{xy} &\to \frac{\ln U_x(k_l)U_y(k_l+\Delta_x)U_x(k_l+\Delta_y)^{-1}U_y(k_l)^{-1}}{\Delta_x\Delta_y} \\ c_n &\to \frac{1}{2\pi i}\sum_l \ln U_x(k_l)U_y(k_l+\Delta_x)U_x(k_l+\Delta_y)^{-1}U_y(k_l)^{-1} \end{aligned} \tag{5}$$

In numerical calculations, the wave function $|n(k_l)\rangle$ is the normalized field distribution of the pressure at the Bloch vector k_l. To save computer memory, we can calculate four points around k_l at one time to obtain the Berry curvature.

It is worth noting that there may exist some degenerate points with unbroken time-reversal symmetry. The equations for the Chern Number mentioned above are invalid at such degenerate points. However, we can further lift this degeneracy by projecting them onto the spin space (two

elastic pseudospins in our model). First, we can calculate the Bloch wave function of our model at the degenerate Γ point, noted as Γ^+ and Γ^-, which can be hybridized as the two normalized pseudospin states $\Gamma^+ \pm i\Gamma^-$. The other wave functions a Bloch vector away from the Γ point can be projected on the two pseudospin basis,

$$\left|n^+\right\rangle = \begin{pmatrix} \left\langle \Gamma^+ \middle| k_+ \right\rangle \\ \left\langle \Gamma^- \middle| k_+ \right\rangle \end{pmatrix}, \left|n^-\right\rangle = \begin{pmatrix} \left\langle \Gamma^+ \middle| k_- \right\rangle \\ \left\langle \Gamma^- \middle| k_- \right\rangle \end{pmatrix} \tag{6}$$

Then, the spin operator can be defined as[6]:

$$s_z = \begin{pmatrix} \left\langle n^+ \middle| \sigma_z \middle| n^+ \right\rangle & \left\langle n^+ \middle| \sigma_z \middle| n^- \right\rangle \\ \left\langle n^- \middle| \sigma_z \middle| n^+ \right\rangle & \left\langle n^- \middle| \sigma_z \middle| n^- \right\rangle \end{pmatrix} \tag{7}$$

We can diagonalize the above equation to obtain the Bloch wave function on the pseudospin space,

$$s_z \left|P^\pm\right\rangle = s_\pm \left|P^\pm\right\rangle \tag{8}$$

Thus, the corresponding Berry curvatures and spin Chern numbers can be obtained as

$$\begin{aligned} F_{xy}^s &\rightarrow \frac{\ln U_x^\pm(k_\pm) U_y^\pm(k_\pm+\Delta_x) U_x^\pm\left(k_\pm+\Delta_y\right)^{-1} U_y^\pm(k_\pm)^{-1}}{\Delta_x \Delta_y} \\ c_n^s &\rightarrow \frac{1}{2\pi i}\sum_l \ln U_x^\pm(k_\pm) U_y^\pm(k_\pm+\Delta_x) U_x^\pm\left(k_\pm+\Delta_y\right)^{-1} U_y^\pm(k_\pm)^{-1} \end{aligned} \tag{9}$$

where $U_\mu^\pm(k_\pm) = \left\langle P^\pm(k_\pm) \middle| P^\pm\left(k_\pm+\Delta_\mu\right)\right\rangle$.

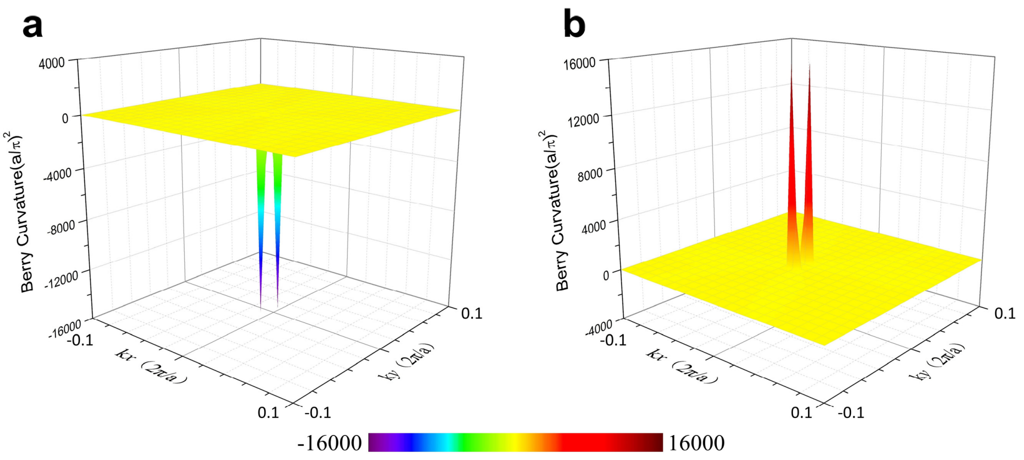

Supplementary Figure 9 | Calculated Berry curvature of the lower two bands (*p* states) in Supplementary Fig. 3f.

Therefore, we can obtain the two independent Berry curvatures for the two elastic pseudospins as shown in **Supplementary Fig. 9**. The nontrivial spin Chern numbers in our model can be obtained via integration of the corresponding Berry curvature over the whole BZ. It should be noted that we did not find singularities in the discrete numerical calculation, although there are some sharp peaks/dips near the original degenerate point. In addition, we also check the trivial case with total zero spin Chern numbers. One can further increase the efficiency to only carry out calculations near the degenerate point(s) because the Berry curvature far from degeneracy is always zero.

Supplementary Note 3:

Shear horizontal modes and their selective inhibition.

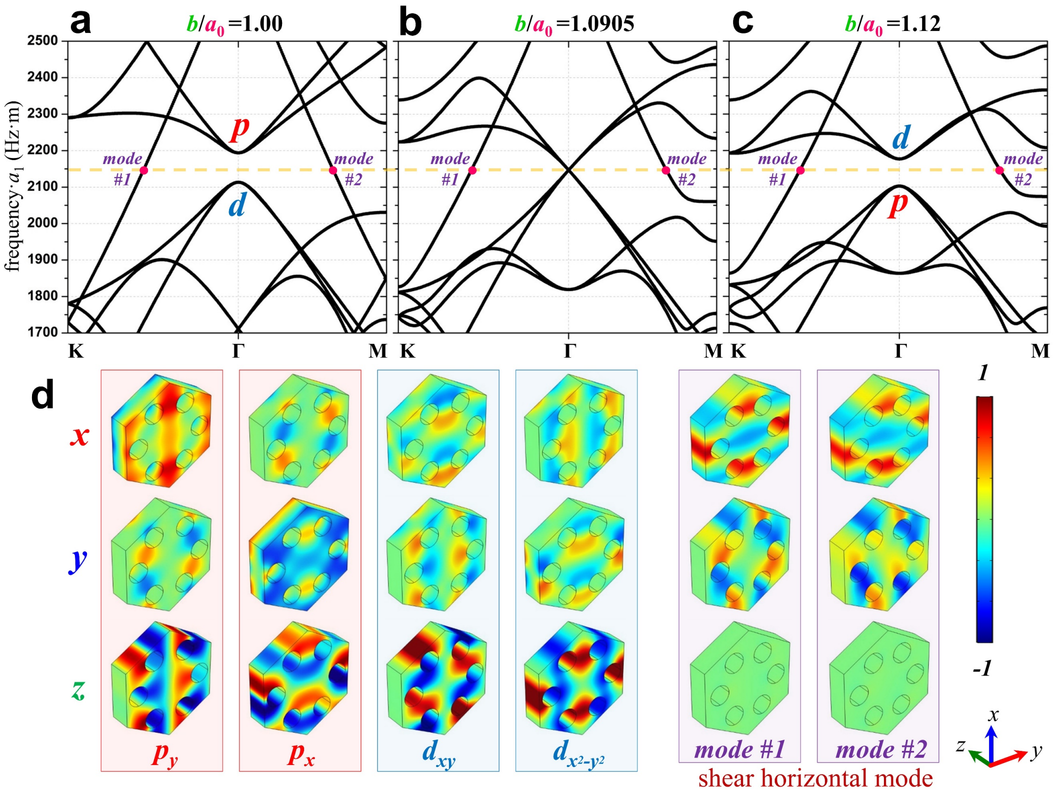

Supplementary Figure 10 | Examination of the primary modes in Supplementary Fig. 3 by

their elastic displacement distributions in the x, y and z directions. Both *p* and *d* modes have elastic displacement components in all three directions. On the contrary, shear horizontal modes, such as *mode#1* and *mode#2*, which are present at the Dirac frequency (yellow dotted line), only have elastic displacement components in the *x* and *y* directions but no components in the out-of-plane *z* direction.

Supplementary Fig. 10a through Supplementary Fig. 10c present the same band diagrams as presented in Supplementary Figure 3 with *b* increasing from $1.00a_0$ (**a**) to $1.0873a_0$ (**b**) and then to $1.12a_0$ (**c**). Supplementary Fig. 10d shows the elastic displacements in the three (x, y and z) directions of all the primary modes in the above band diagrams. Clearly, both the p and d modes have elastic displacement components in all three directions. Except for the four dispersions, which form the double Dirac degeneracy, there are still two dispersions (e.g., *mode#1* and *mode#2* present at the Dirac frequency). These dispersions consist of shear horizontal (SH) modes that only have components in the *x and y* directions but no components in the out-of-plane z direction. SH modes and other modes in the plate (like the *p* and *d* modes) are intrinsically different and can be independently handled both in theory and experimentally: at the theoretical level, the topological nature of the SH bands and the *p*/*d* bands are irrelevant; at the experimental level, the SH modes and the *p*/*d* modes can also be independently excited (or inhibited).

The SH modes, as the grey dispersions shown in Supplementary Fig. 3 (and in Fig. 1b of the main text), can be selectively inhibited by utilizing (out-of-plane) z-polarized transducers, *e.g.*, one can attach piezoelectric longitudinal-wave transducers to the plate surface or use interdigital transducers (IDTs) on piezoelectric materials at specific orientations.

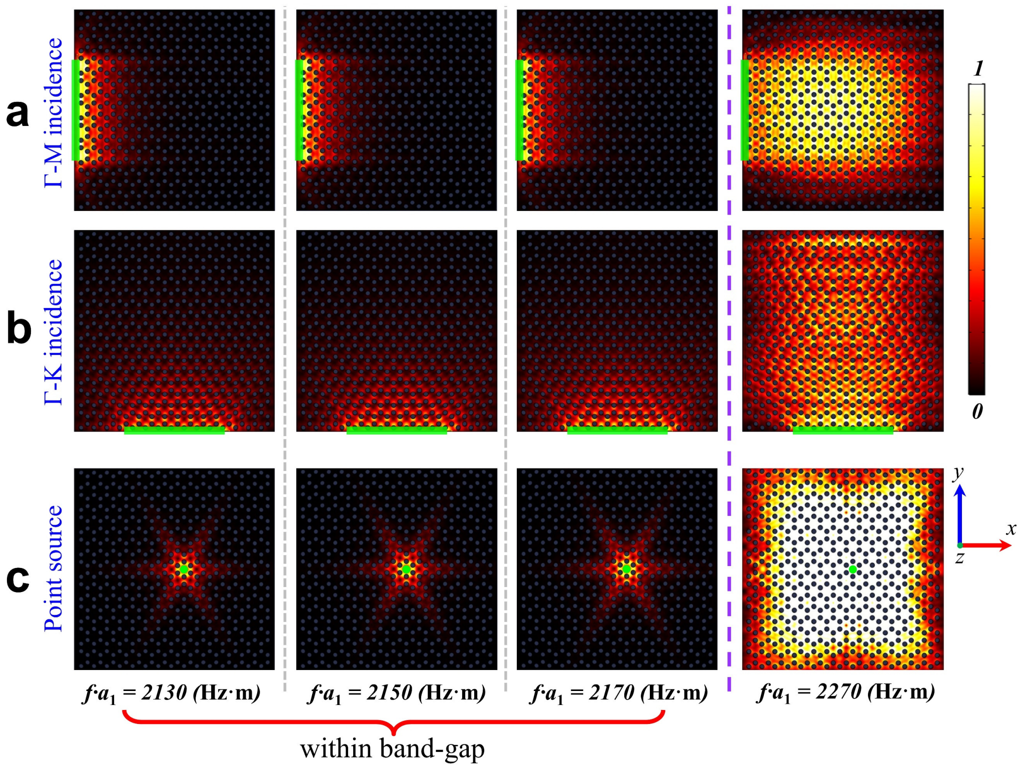

Supplementary Figure 11 | Selective inhibition of the shear horizontal mode: Calculated elastic, total displacement field when (out-of-plane) z-polarized transducers, as the green lines/points indicate, are applied in three different cases: (**a**) Γ-M incidence to the plate phononic crystal (same as in Supplementary Figure 3a), (**b**) Γ-K incidence and (**c**) inside the plate phononic crystal. Four frequencies are examined: three in the bulk band-gap and one off (above) the bulk band-gap (ignoring the SH dispersion).

Supplementary Fig. 11 shows the calculated (root-mean-square, RMS) total displacement fields inside the plate phononic crystal of Supplementary Fig. 3a when we apply a z-polarized transducer as (**a**) a line source for Γ-M incidence, (**b**) a line source for Γ-K incidence and (**c**) a point source placed in the centre of the phononic crystal at different frequencies in and off (above) the "bulk band-gap" (ignoring the SH modes). Clearly, when the operation frequency is in the "bulk band-gap", there are no mode excitations in all of these cases. Therefore, the SH modes are indeed inhibited.

Supplementary Note 4:

Integratable elastic phononic circuits with backscattering immunity

The topological features demonstrated in our paper can be exploited for promising chip-scale surface acoustic wave (SAW) applications. In particular, backscattering-suppressed SAW transport that is robust against various defects and geometry distortions relaxes many design restrictions, enabling SAW phononic circuits with arbitrary geometries while simultaneously allowing a superior performance. Backscattering-suppressed SAW transport allows for a whole new range of topological electro-(opto-) mechanical devices that can be readily implemented on other monolithic platforms including AlN, $LiNbO_3$ or Si substrates.

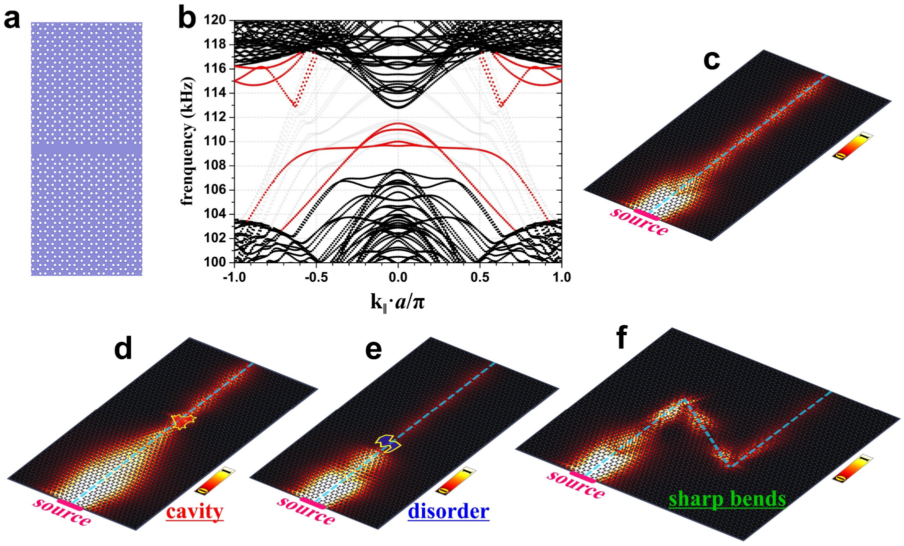

Supplementary Figure 12 | Elastic topological trivial waveguide. (**a**) By simply moving two rows in the phononic crystal of Supplementary Fig. 3a, an original elastic waveguide is constructed. (**b**) Projected band diagram of this elastic waveguide. The edge states (red dispersions) are topologically trivial, and hence, they are always gapped. Panels **c** through **f**: Calculated elastic distribution fields for four different cases of this waveguide; (**c**) no defect, (**d**) defect of an arbitrarily-shaped cavity without perforated holes, (**e**) defect of an arbitrarily-shaped disorder of randomly ordered perforated holes, and (**f**) a Z type bend. Large back-scattering can be clearly observed in all three cases.

In comparison to the TI-OI interface for the elastic waveguiding demonstrated in our main text,

an original waveguide made from an OI-OI interface is constructed for a contrast experiment. As shown in **Supplementary Fig. 12**, the results for similar defects and bends are drastically different. All the defects will cause distinct elastic resonances, while the bends will severely inhibit the elastic forward propagation, leading to a decreased transmission or even a total reflection.

In regard to the scale sizes and working frequencies, our scheme is feasible as long as the total number of material atoms/molecules in the whole sample can still be considered sufficiently large. At this point, the local nanoscopic properties of the material atoms/molecules are still unclear and the whole sample can still be characterized by its global dynamical behaviours, which are described by a classical, linear, elastic theory. In this theoretical framework, all elastic parameters have no relationship with the working frequency (in contrast with electromagnetic waves). Although it is difficult to determine the exact transition point where the classical elastic theory becomes invalid, at the very least, this theory should be valid for systems in which the lattice constants are larger than 100 nm (i.e., 10^7~10^9 atoms in one unit-cell). As the lattice constant becomes smaller, the wavelength of the elastic wave affected by the phononic crystal needs to be scaled (linearly) to smaller values. Since the velocity of the elastic wave depends on only the elastic parameters, which have no relationship with the frequencies, the velocity of the elastic wave is frequency-independent, which further implies that, in our system, as the lattice constant becomes smaller, the working frequency of the elastic wave should be scaled (linearly) to higher values. Hence, as shown in **Supplementary Fig. 13** (chip-scale silicon case), the vertical axis is "frequency • lattice constant (Hz • m)". For instance, if the lattice constant of the Silicon phononic crystal is 1 um, the frequency of the helical edge states will be approximately 3.5 GHz. Of course, for different kinds of materials, this frequency will slightly vary according to the elastic parameters of the materials; however, the variation of the frequency will barely exceed one order of magnitude.

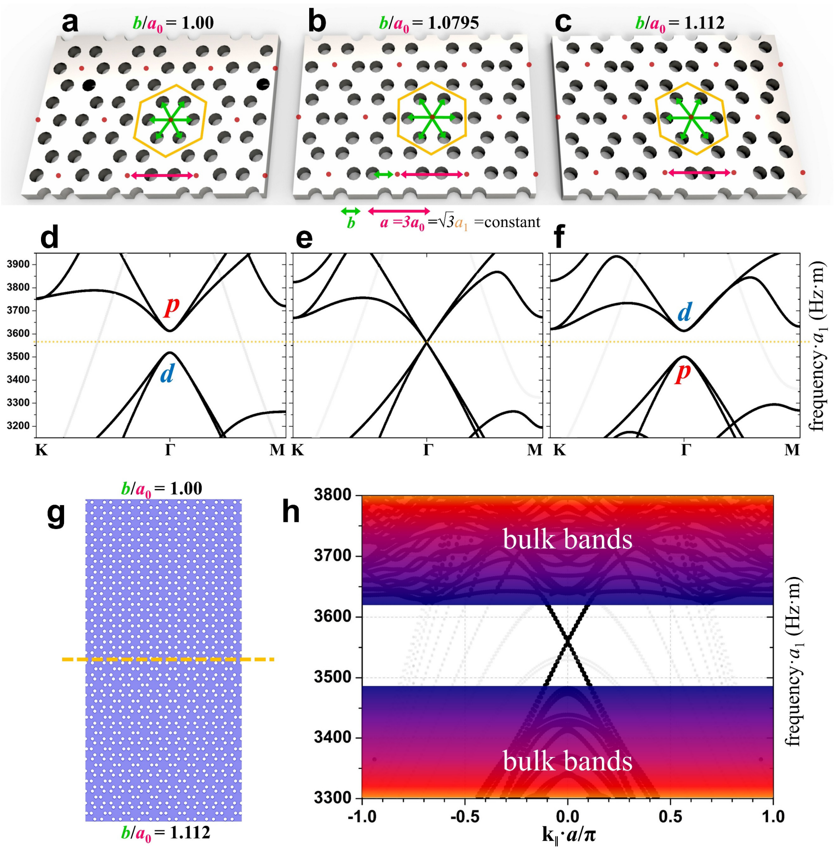

Supplementary Figure 13 | Chip-scale Silicon (Si) case. Panels (**a**) through (**c**): Geometrical evolution, and (**d**) through (**f**) are the corresponding bulk band diagrams. The lattice constant a (a=3a_0= √3a_1), thickness of the Si plate (0.4a_1), and radius of perforated holes r (0.18a_1) are constant. When the hole-centre distance, b, increases from (**a**) 1.00a_0 to (**b**) 1.0795a_0 and then to (**c**) 1.112a_0, band inversion of the p/d modes occurs in an elastic insulator (ignoring the grey SH modes). (**g**) Elastic waveguide constructed by attaching the Si phononic crystal in a and c. (**h**) Projecting band structure showing the elastic helical edge states ranging from 3480 to 3620 Hz • m.

Regarding substrate materials and SAWs transduction, due to the large impedance discontinuities that are common to the interface between almost any solid material and air, surface elastic waves can always be supported at the interface. Therefore, our approach is always effective and can be generally applied to almost all solid-state materials whether they are macro-scale materials (such as stainless steel, aluminium alloy, glass, etc.), integrated CMOS/ MEMS materials (such as Si, SiC, etc.), or chip-scale piezoelectric materials (such as $LiNbO_3$, $LiTaO_3$, quartz, AlN, ZnO, etc.). For a specific substrate, the geometric parameters, *i.e.*, r (radius of the holes) and b

(distance between the holes to the centre of the unit-cell), need to be carefully tailored.

For miniaturized and integrated systems working at high frequencies, there are mature SAW transducer solutions for both non-piezoelectric or piezoelectric substrates. For piezoelectric substrates (such as $LiNbO_3$), SAWs can be excited or received by means of interdigital transducers (IDTs), *i.e.*, finger-like metal electrodes. For non-piezoelectric materials (such as Si), the IDTs are still applicable by pre-depositing a thin layer of piezoelectric material (such as ZnO) on the original substrate. The transducer frequencies depend on both the SAW velocities of different substrate materials and the finger width of the IDTs. At present, SAWs can be easily transduced to several GHz and beyond.

Regarding the mechanical losses of solid-state elastic phonons, the intrinsic mechanical losses in acoustic systems mostly come from viscous dissipation, heat conduction, and molecular relaxation. Among these, viscous dissipation is dominant, and its value is proportional to the square of the acoustic frequency. In systems of fluid airborne sound, the viscous dissipation is considerable even at sonic frequencies. For instance, in an ordinary atmosphere (temperature of 20℃, standard pressure and 50% humidity), the viscous dissipation of a 10 kHz sound is approximately 160 dB/km^{-1} (or an approximate 5 dB loss for every 1000 wavelengths of propagation)[7]. At higher frequencies, for instance, an ultrasonic (100 kHz) wave, this value increases to 3300 dB/ km (that is, an approximate 10 dB loss for every 1000 wavelengths of propagation). Consequently, airborne sounds with higher frequencies cannot be used at workplaces because of the distinct viscous dissipation. This situation is similar for fluid water sounds.

On the contrary, in solid-state acoustic systems, as in our present work, the viscous dissipation is much smaller. For instance, in the most widely used substrate material for SAW devices, *e.g.*, $LiNbO_3$, the viscous dissipation is only 0.35 dB/μs even at a much higher frequency of 1 GHz (that is, there is an approximate 0.35 dB loss for every 1000 wavelengths of propagation)[8]. Considering that the sizes of SAW devices are only roughly tens of SAW wavelengths, the loss is almost negligible. In our solid-state acoustic experiments, similar to the waveguides that demonstrate the backscattering immunity in Fig. 3 of the main text, the working frequencies are only tens of kHz and

the samples are only tens of wavelengths in size. Thus, we can by no means measure any dissipation experimentally. According to a rough estimation, the length of our topological waveguide should reach up to 10^3~10^5 times our present value before the amplitude decays by half. Even if our system is scaled to GHz, this value is still 10^2~10^4 times the present one (in a miniaturized device).

Regarding the high energy flux/capacity of the elastic waveguide, in experiments, the energy fluxes flowing through our TI-OI interfaces depend on the power loaded on our ultrasonic transducers and the transducing efficiency into our edge modes. Practically, this value can be estimated from a combination of experiment and numerical simulation (finite element method by COMSOL Multiphysics: Structural Mechanics Module under linear elasticity) by following these steps: **First**, experimentally measure the surface out-of-plane displacement in the sample. For example, the measured amplitude of the surface displacement at a particular point (x, y, z_0) near the TI-OI interface is $A_{\mathrm{exp}}(\mathrm{x,y,z_0})$. This value can be up to tens of nanometres in the experiments. **Second**, numerically adjust the power of the acoustic source in the simulation until the simulated amplitude of the surface displacement at the same point (x, y, z_0) is equal to the experimentally measured one, *i.e.*, $A_{\mathrm{sim}}(x, y, z_0)=A_{\mathrm{exp}}(x, y, z_0)$. **Third**, according to the second step, the numerical energy fluxes at each point $F_{\mathrm{sim}}(x, y, z)$ are now fixed. Then, the total energy flux transfer through the edge can finally be estimated as the surface integral, $\int F_{\mathrm{sim}}(x, y, z)\mathrm{d}y\mathrm{d}z$.

By following the above steps, the energy fluxes flowing through the TI-OI interface are estimated to be roughly several tens of watts (J/s). Note that, of course, this value is variable since the loading power of the ultrasonic transducer in the experiments is adjustable. Theoretically, the upper limit of the energy flux depends on the elastic energy capacity of the solid-state substrate material. According to the theory of linear elasticity, the elastic energy capacity depends on the maximum strain as long as the linear stress-strain relation is valid. Following the same steps as in the above estimation of the energy flux, one changes the power of the acoustic source in the simulation until the simulated stress at the maximum displacement point near the TI-OI interface (this point can be easily found in our simulation) is equal to the maximum stress within the linear stress-strain relation. Finally, the upper limit of the energy flux can be roughly estimated to be over 10^8 watts (J/s). In miniaturized cases, on Si, $LiNbO_3$ or an AlN substrate operating at hundreds of MHz to several

GHz, this value could be over 10^{-4} watt (J/s). Consequently, in solid-state systems, all these values are much higher than in other fluid acoustic media. Along with the very low losses in the elastic systems, their dynamic ranges will be even larger (by orders of magnitude) for advanced signal processing. Moreover, intense wave-matter interactions can also be expected for reinforcing optomechanics and quantum acoustics.

Supplementary Note 5:

Pseudospin-selective waveguide coupler (*i.e.*, elastic beam splitter)

Generally, it is not easy to selectively excite a particular pseudospin in an experiment, even with multiple excitation sources. Here, we experimentally design a pseudospin-selective waveguide coupler that allows us to distinguish and separate pseudospin-dependent transport with a very high fidelity, even with an unknown spin excitation.

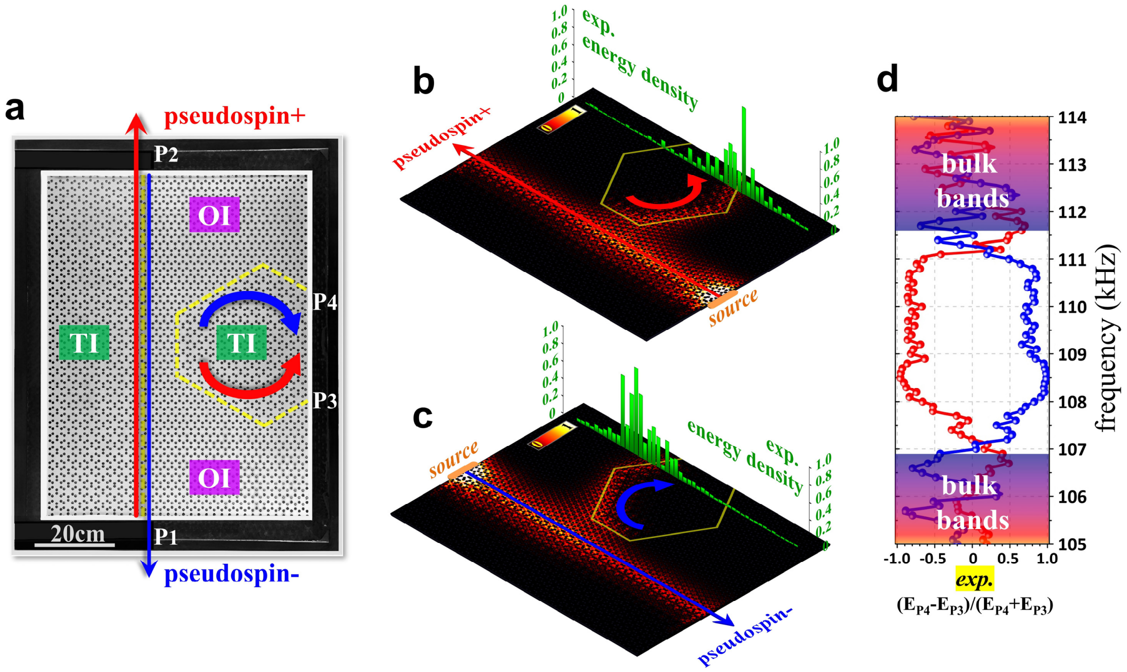

Supplementary Figure 14 | Pseudospin-selective waveguide coupler. (**a**) Photograph of an elastic waveguide-coupler to demonstrate elastic one-way pseudospin-dependent transport. The

whole sample is divided into three sections by a sandwich-like structure composed of an elastic ordinary insulator (b=1.00a_0) between two elastic topological insulators (b=1.12a_0). Thus, two topologically protected elastic waveguides are formed: a straight waveguide on the left bounded by a partial hexagonal waveguide on the right, as the yellow dotted lines indicate. The two ports of the straight waveguide are labelled as P1 and P2, and the two ports of the partial hexagonal waveguide are labelled as P3 and P4. In this experiment, when the coupling of the two waveguides occurs, only anti-clockwise (clockwise) circulating propagation along the partial hexagonal waveguide is allowed for elastic pseudospin+ (pseudospin−) modes, as indicated by the red (blue) circular arrow. Panels (**b**) and (**c**): (bottom) Heat maps are calculated elastic energy-density fields at a given frequency (108.5kHz) within the bulk band-gap. (top) Green bars are experimental results that are spatially measured along a straight line near both P3 and P4 of the partial hexagonal waveguide. In both the calculation and experiment, we applied ultrasonic longitudinal-wave transducers attached to the sample surface at P1 and P2 to excite only the elastic pseudospin+ and pseudospin− modes, respectively. (**d**) Experimentally measured spectra of the contrast ratio of the elastic energy-density between P3 and P4, defined as (EP3-EP4)/(EP3+EP4), for elastic pseudospin+ (red) incidence from P1 and for pseudospin− (blue) incidence from P2. Shadow regions correspond to the bulk bands.

As shown in **Supplementary Fig. 14a**, our waveguide coupler is divided into three sections by a sandwiched structure composed of TI and OI. Hence, two topological waveguides are formed: a straight waveguide on the left bounded by a partial hexagonal waveguide on the right. There are four input/output ports in the whole waveguide coupler. The two ports in the straight waveguide are labelled P1 and P2, and the two ports in the partial hexagonal waveguide are labelled P3 and P4. Coupling of nearby waveguides can generally happen if both waveguides support the same eigenmodes. In the coupling of two ordinary waveguides, the direction of wave propagating from one waveguide to the other will be chosen principally as the direction of original energy flow. For example, if the energy initially flows from bottom to top along the straight waveguide, when the wave is transporting near the coupling area of the two waveguides, the energy will instinctively flow into the hexagonal waveguide following its original direction, *i.e.*, flow into the upper half of the hexagonal waveguide in a clockwise direction. Conversely, if the original energy flow is from top to bottom in the straight waveguide, the energy will flow into the lower half of the hexagonal waveguide in an anti-clockwise direction after the coupling occurs. However, in a

pseudospin-dependent waveguide, though both pseudospin+ and pseudospin− modes are supported, each can only flow along a unique direction. Put simply, when one places a transducer near one port of the waveguide, only the definite pseudospin+ or pseudospin− mode can be excited and propagate through the waveguide. For example, if a transducer was placed near the bottom (top) port of the straight waveguide, P1 (P2), only the elastic pseudospin+ (pseudospin−) mode can be excited and transmitted through the waveguide with respect to its transmission direction, *i.e.*, TI is located on the left (right) side, while OI is located on the right (left) side. In this circumstance, because both the elastic pseudospin+ and pseudospin− modes are preserved in the coupling of the two waveguides and no spin inversion mechanism is involved in the whole configuration, only the anti-clockwise (clockwise) propagation of elastic waves in the pseudospin+ (pseudospin−) mode will appear in the hexagonal waveguide in the end, which implies that the energy will no longer flow along its original direction after passing from one waveguide to the other.

Elastic energy distributions by both finite element method (FEM) simulation and practical experimentation with a longitudinal wave transducer attached to the sample substrate near P1 and P2 are shown in Supplementary Fig. 14b and Supplementary Fig. 14c, respectively. At an ultrasonic frequency in the bulk band-gap, it is clear that there is only one elastic pseudospin in the clockwise or counterclockwise direction that can be detected in the hexagonal waveguide. Such observations are consistent with our theoretical expectations. Moreover, spectra of the energy densities near both ports P3 and P4 (defined as EP3 and EP4) are also measured. Their contrast ratios, defined as (EP3-EP4)/(EP3+EP4), are shown in Supplementary Fig. 14d confirming a high contrast energy flow within the bulk band-gap frequency where the waveguide coupling occurs. All these experimental observations clearly confirmed the elastic, one-way, pseudospin-dependent transport.

Another important example of this is a monolithically integrated spin splitter that allows us to separate/distinguish the spin-dependent transport with a very high fidelity, even in the case of an unknown spin excitation. To illustrate this, a four-port, topologically protected device was constructed using TI and OI as shown in **Supplementary Fig. 15a**.

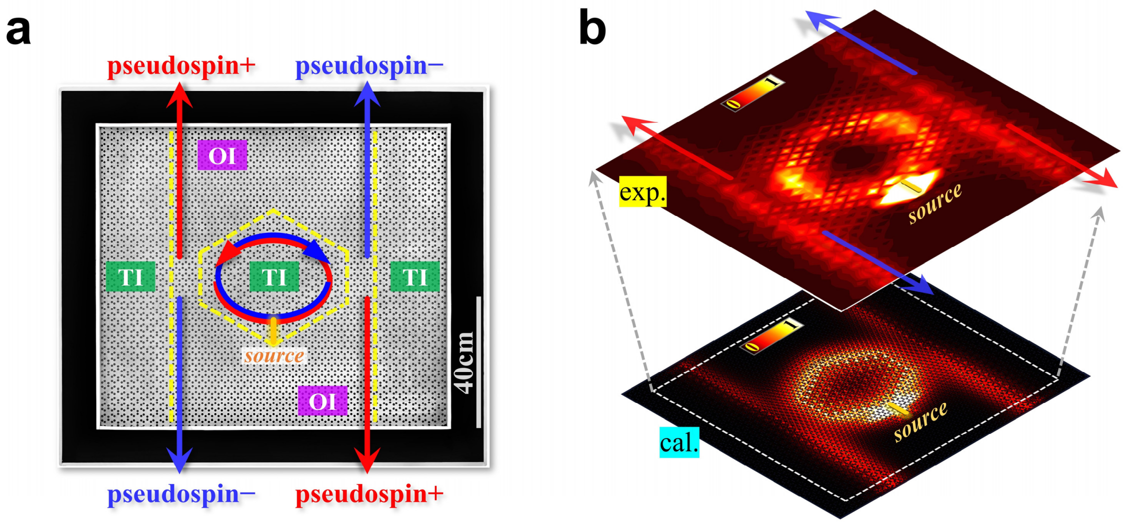

Supplementary Figure 15 | Four-port, topologically protected spatial pseudospin splitter. (**a**) Photo of a topologically protected, four-port device constructed with one OI and three TIs. Three topological interfaces exist as indicated by the yellow dashed lines, which represent straight waveguides on the left and right side and a hexagonal resonator between them. A transducer is placed at the middle bottom section of the hexagonal resonator to excite the (red) anti-clockwise elastic pseudospin+ mode and/or (blue) clockwise pseudospin− mode inside the resonator. (**b**) (bottom) Calculated and (top) experimentally imaged elastic energy-density distribution. Because of spin-momentum locking, the elastic pseudospin+ mode and/or pseudospin− mode can be perfectly separated and distinguished (according to the analysis in Supplementary Figure 14) based on the outgoing ports.

This splitter consists of two straight waveguides with one hexagonal resonator (same as in Fig. 3a of the main text) between them, coupling the four-ports as an integrated circuit. Now, if the transducer is placed in the middle bottom of the hexagonal resonator, a mixed pseudospin state can be excited with elastic pseudospin+ and pseudospin− modes circulating anti-clockwise and clockwise in the hexagonal resonator, respectively. These pseudospin states will circulate in the hexagonal resonator, and a portion of them will couple to the four outgoing ports, as illustrated by the obtained normalized elastic energy distribution in Supplementary Fig. 15b. Clearly, because of spin-momentum locking, only the pseudospin+ (pseudospin−) mode can couple to and be transmitted through the straight waveguide in its preferred path into the lower-left (upper-left) and upper-right (lower-right) ports. This spin-sorting functionality basically constitutes a spatial pseudospin-splitter.

Supplementary Note 6:

Additional amplitude/phase information of the elastic pure pseudospin current.

In the experiment presented in Figure 4 of the main text, two transducers are deliberately set with reversed phases (0° vs. 180°) such that the whole experimental setup (including the TI-OI interface and the two working transducers) is totally anti-symmetric in both real space and the time-domain along the middle plane of the elastic waveguide. Consequently, the superposition of elastic pseudospin+ and pseudospin− modes in the middle area of the sample will necessarily present an anti-symmetric pattern in both space and time, *i.e.*, the constant $+A \leftrightarrow -A$ single polarization in the pseudospin space.

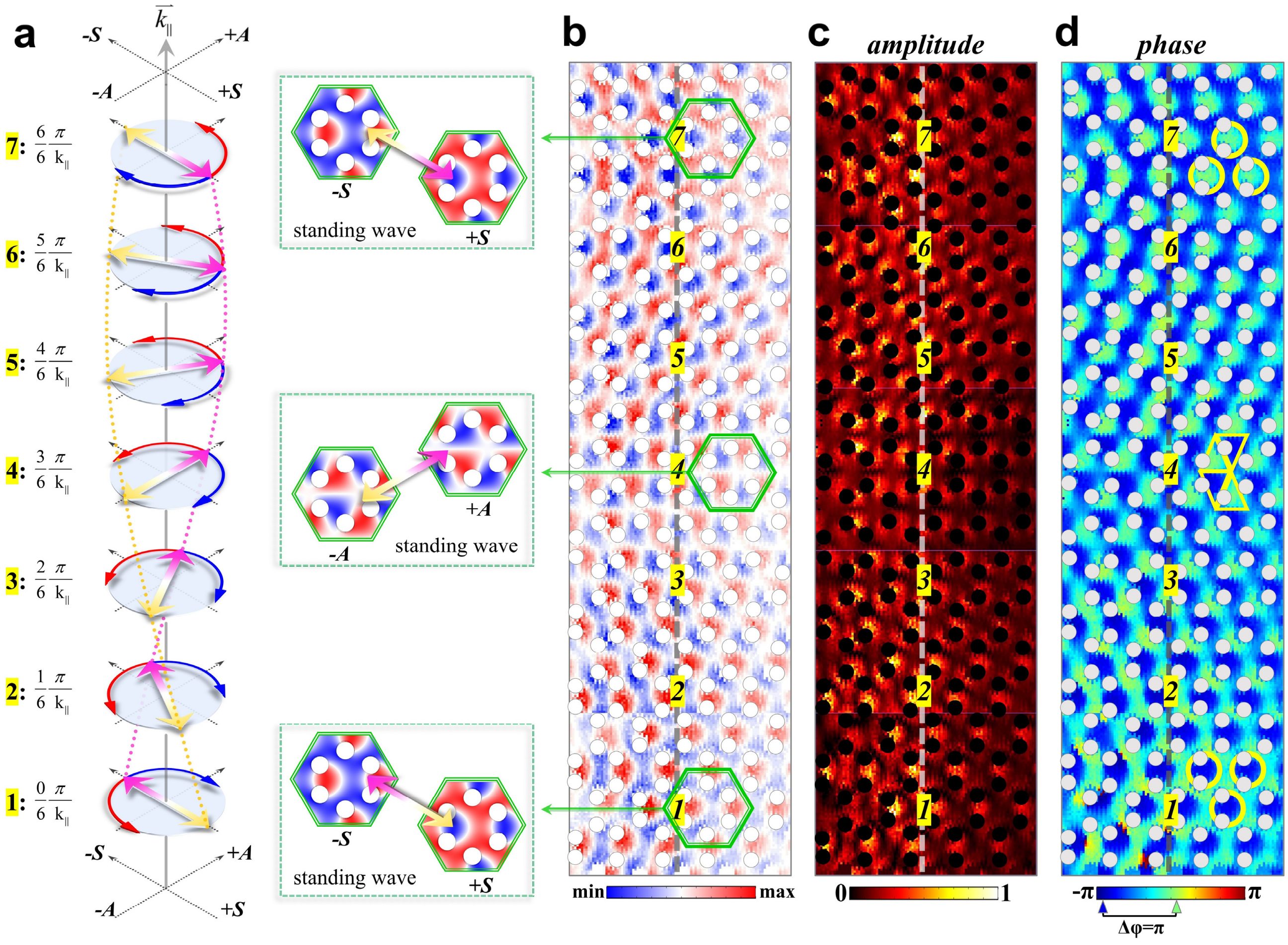

Supplementary Figure 16 | Elastic spin current without energy flow. (**a**) Interference of pseudospin+ and pseudospin− modes in the middle (green) region of Fig. 4a of the main text.

Experimentally measured elastic (**b**) field distribution, (**c**) amplitude and (**d**) phase of the out-of-plane displacement characterizing the pseudospin transport along the TI-OI interface.

In addition to the measured out-of-plane displacement distribution field presented in **Supplementary Fig. 16b** (same as Fig. 4c of the main text), the detailed amplitude and phase distribution fields shown in Supplementary Fig. 16c and Supplementary Fig. 16d further confirmed our theoretical expectations: 1) The variation of the phases is discontinued in platform islands with a near consistent step difference of 180 °, and thus, the boundaries of these islands (as the yellow closed lines indicate in Supplementary Fig. 16d) are indeed the "wave nodes" in the two-dimensional space indicating the elastic pseudospin current's standing wave nature without elastic energy flow. 2) The amplitude demonstrates a "symmetric—anti-symmetric—symmetric" pattern with spatial evolution along the waveguiding direction ($\overline{k_{\parallel}}$), while the phase shows a more clear "(+)symmetric—anti-symmetric—(−)symmetric" pattern indicating the standing pseudospin current helical evolution in real space.

Supplementary References:

[1]. Wu, L.-H. & Hu, X. Scheme for achieving a topological photonic crystal by using dielectric material. *Phys. Rev. Lett.* **114**, 223901 (2015).

[2]. Mousavi, S. H., Khanikaev, A. B. &Wang, Z. Topologically protected elastic waves in phononic metamaterials. *Nat. Commun.* **6**, 9682 (2015).

[3]. Yu, S.-Y., et. al. Surface phononic graphene. *Nature Maters.* **15**, 1243 (2016).

[4]. Laerme, F., et al. Bosch deep silicon etching: improving uniformity and etch rate for advanced MEMS applications. Micro Electro Mechanical Systems, 1999. MEMS'99. Twelfth IEEE International Conference on. IEEE, (1999).

[5]. T. Fukui, Y. Hatsugai, H. Suzuki, Chern numbers in discretized Brillouin zone: Efficient method of computing (spin) Hall conductances, *J. Phys. Soc. Jpn.* **74**, 1674-1677 (2005).

[6]. E. Prodan. Robustness of the spin-Chern number, *Phys. Rev. B* **80**, 125327 (2009).

[7]. The speed and attenuation of sound:
http://www.kayelaby.npl.co.uk/general_physics/2_4/2_4_1.html

[8]. Damon, R. W. Solid-state microwave delay lines. *IEEE spectrum*, **4**(6), 87-92 (1967).
