# Supplementary material for: Elastic pseudospin transport for integratable topological phononic circuits
Source: Nat Commun. 2018 Aug 6;9:3072. doi: 10.1038/s41467-018-05461-5 (PMC6078995; doi:10.1038/s41467-018-05461-5)
Supplement: Supplementary file 2 — Description of Additional Supplementary Files [file 41467_2018_5461_MOESM2_ESM.pdf]

Description of Additional Supplementary Files

File Name: Supplementary Movie 1
Description: Time-dependent elastic field distribution of an elastic pseudospin+ (S+iA).

File Name: Supplementary Movie 2
Description: Time-dependent elastic field distribution of an elastic pseudospin－(S－iA).

File Name: Supplementary Movie 3
Description: Time-dependent elastic field distribution of an elastic pure pseudospin current without energy flow.
